# A new approach to weighted Sobolev spaces

**DOI:** 10.1007/s00605-024-02044-z

**Published:** 2025-01-09

**Authors:** Djameleddine Kebiche

**Affiliations:** Faculty of Mathematics, University of Vienna, Oskar-Morgenstern-Platz 1, 1090 Wien, Austria

**Keywords:** Weighted Sobolev spaces, Degenerate elliptic PDEs, Poincaré inequality, 46E35, 35J70, 35A23

## Abstract

We present in this paper a new way to define weighted Sobolev spaces when the weight functions are arbitrary small. This new approach can replace the old one consisting in modifying the domain by removing the set of points where at least one of the weight functions is very small. The basic idea is to replace the distributional derivative with a new notion of weak derivative. In this way, non-locally integrable functions can be considered in these spaces. Indeed, assumptions under which a degenerate elliptic partial differential equation has a unique non-locally integrable solution are given. Tools like a Poincaré inequality and a trace operator are developed, and density results of smooth functions are established.

## Introduction

By a weight function $u:\Omega \longrightarrow \mathbb {R}$ we mean a locally integrable function which is non-negative almost everywhere in $\Omega$. For $p\in [1,\infty )$, $L^{p}(\Omega ,u)$ denotes the space of all functions $f:\Omega \longrightarrow \mathbb {R}$ satisfying $fu^{(1/p)} \in L^p(\Omega )$. Let $m\in \mathbb {N}$ and consider a collection $S:=\{u_{\alpha }\}_{\alpha \in \pi _{m}}$ ($\pi _{m}:=\{\alpha \in \mathbb {N}^{d}\mid |\alpha |\le m$}) of weight functions on $\Omega$. The weighted Sobolev space $W^{m,p}(\Omega ,S)$ is defined as the space of all functions $f\in L^{p}(\Omega ,u)\cap L_{\textrm{loc}}^{1}(\Omega )$ such that their distributional derivative are elements of $L^{p}(\Omega ,u_{\alpha })\cap L_{\textrm{loc}}^{1}(\Omega )$. The space $W^{m,p}(\Omega ,S)$ is equipped with the norm

$$\begin{aligned} \forall f\in W^{m,p}(\Omega ,S):\,||f||_{W^{m,p}(\Omega ,S)}:=\left( \sum _{|\alpha |\le m}||u_{\alpha }^{1/p}D^{\alpha }f||_{L^{p}}^{p}\right) ^{1/p}. \end{aligned}$$

A sufficient condition for the space $W^{m,p}(\Omega ,S)$ to be a Banach space is

1.1 $$\begin{aligned} \forall \alpha \in \pi _{m}:\,u_{\alpha }^{-1}\in L_{\textrm{loc}}^{\frac{1}{p-1}}(\Omega ) \end{aligned}$$

(see Thm. 1.11 of [5]). Indeed, the assumption (1.1) implies that for all $\alpha \in \pi _{m}$, the space $L^{p}(\Omega ,u_{\alpha })$ is continuously embedded into $L_{\textrm{loc}}^{1}(\Omega )$, and the functional $W^{m,p}(\Omega ,S)\longrightarrow \mathbb {R}$, $f\longrightarrow \int _{\Omega }f\partial ^{\alpha }\varphi \textrm{d}x$ ($\varphi \in {\mathcal {D}}(\Omega )$) is continuous, a crucial property used in the proof of the completeness of the space $W^{m,p}(\Omega ,S)$ (see Remark 1.8 of [5]). However, when (1.1) is violated, the weighted Sobolev space $W^{m,p}(\Omega ,S)$ is not necessary a Banach space (see Example 1.12 of [5]). This is because, when (1.1) is violated, the latter functionals are, in general, no longer continuous. The existing remedy consists in replacing the set $\Omega$ with a smaller one so that the property (1.1) holds. More precisely, the new set, which will be denoted by $\Omega _{S}^{*}$, is defined by $\Omega _{S}^{*}:=\Omega \backslash \cup _{\alpha \in \pi _{m}}P(u_{\alpha })$ where $P(u_{\alpha })$ is the set of points $x\in \Omega$ for which $u_{\alpha }^{-1}\not \in L^{\frac{1}{p-1}}(\Omega \cap U)$ for all neighborhood *U* of *x*. The set $B:=\cup _{\alpha \in \pi _{m}}P(u_{\alpha })$ is closed (and hence $\Omega _{S}^{*}$ is open) and Lebesgue negligible. Moreover, property (1.1) holds when $\Omega$ is replaced with $\Omega _{S}^{*}$ and hence the weighted Sobolev space $W^{m,p}(\Omega _{S}^{*},S)$ is a Banach space (see Sec. 3 of [5] for more details). However, the disadvantage of this remedy is a modification of the domain. As a consequence, the convexity might be lost and the boundary of $\Omega$ is changed.

The alternative we present in this paper consists in replacing the notion of distributional derivative with a new notion of weak derivative, called weak derivative in the sense of $L_{v,\textrm{loc}}^{1}(\Omega )$ (*v* being a ${\mathcal {C}}^{m}(\Omega )$ function). It consists in replacing the space of test functions, which is ${\mathcal {C}}_{c}^{m}(\Omega _{S}^{*})$ in the case of $W^{m,p}(\Omega _{S}^{*},S)$, with a larger one whose elements do not necessary vanish near the set *B* (see Def. 4). When the function *v* is well chosen, i.e. $\Omega _{S}^{*}=\Omega _{|v|^{mp+p}}^{*}:=\Omega \backslash P(|v|^{mp+p})$, the two notions of derivative coincide (see Proposition 7).

In Sect. 5, several density results of smooth functions in weighted Sobolev spaces are proved. In Sect. 6, we give sufficient conditions under which (continuous) trace operators can be defined. A Poincaré inequality is established in Sect. 8. In the last section, we deal with degenerate elliptic linear partial differential equations, where in particular we give assumptions under which a solution is not locally integrable.

## The weighted Lebesgue space $L_{w}^{p}(\Omega )$

Throughout this paper, a weight function $u:\Omega \longrightarrow \mathbb {R}$ is a locally integrable function, which is positive a.e. in $\Omega$. The set of all the weight functions on $\Omega$ is denoted by $W(\Omega )$.

We now give a definition of weighted Lebesgue spaces (using notations slightly different from the classical ones)

### Definition 1

Let $\Omega \subseteq \mathbb {R}^{d}$ be an open set, $p\in [1,\infty )$, and let *w* be such that $|w|^{p}\in W(\Omega )$ (in the sequel we write simply $w\in E_{p}(\Omega )$). We define the weighted Lebesgue space $L_{w}^{p}(\Omega )$ as the space of measurable functions *f* on $\Omega$ such that $fw\in L^{p}(\Omega )$. This space will be equipped with the norm $||\cdot ||_{L_{w}^{p}}$ defined by

2.1 $$\begin{aligned} \forall f\in L_{w}^{p}(\Omega ):\,||f||_{L_{w}^{p}(\Omega )}:=||fw||_{L^{p}(\Omega )}. \end{aligned}$$

The space $L_{w}^{2}(\Omega )$ is equipped with the scalar product $(\cdot ,\cdot )_{L_{w}^{2}}$ defined by

2.2 $$\begin{aligned} \forall f,g\in L_{w}^{2}(\Omega ):\,(f,g)_{L_{w}^{2}}:=(wf,wg)_{L^{2}} \end{aligned}$$

where $(\cdot ,\cdot )_{L^{2}}$ is the usual scalar product of the space $L^{2}(\Omega )$.

Moreover, we write $f\in L_{w,\textrm{loc}}^{p}(\Omega )$ if $f\in L_{w}^{p}(K)$ for all $K\Subset \Omega$.

If we denote by $L^{p}(\Omega ,u)$ the weighted Lebesgue space with the weight function *u*, then we clearly have

$$\begin{aligned} L_{w}^{p}(\Omega )=L^{p}(\Omega ,|w|^{p}). \end{aligned}$$

The reason for using *w* instead of the weight function $|w|^{p}$ is purely for notational reason.

The following theorem is well known (see e.g. Thm. III. 6.6 of [2]).

### Theorem 2

Let $\Omega \subseteq \mathbb {R}^{d}$ be an open set, $w\in E_{p}(\Omega )$, and $p\in [1,\infty )$. Then, the space $L_{w}^{p}(\Omega )$ equipped with the norm (2.1) is a Banach space (Hilbert space when $p=2$).

Let ${\mathcal {S}}$ be the Schwartz space. For a fixed non-null polynomial *P* we set

$$\begin{aligned} {\mathcal {S}}_{P}=\{\psi \in {\mathcal {S}}:\,\psi =P\varphi ,\varphi \in {\mathcal {S}}\} \end{aligned}$$

which is a linear subspace of ${\mathcal {S}}$ equipped with the sequence $(||\cdot ||_{k})$ of semi norms of ${\mathcal {S}}$. Having denoted by ${\mathcal {S}}'_{P}$ the dual space of ${\mathcal {S}} _{P}$, we have the following proposition.

### Proposition 3

Let *P* be a non-null polynomial function and let $p\in [1,\infty )$. Then, any element $f\in L_{P}^{p}(\mathbb {R}^{d})$ defines an element of ${\mathcal {S}}'_{P}$ in following way

$$\begin{aligned} \forall \psi \in {\mathcal {S}}_{P}:\,\langle f,\psi \rangle _{{\mathcal {S}}'_{P},{\mathcal {S}} _{P}}:=\int _{\mathbb {R}^{d}}f(x)\psi (x)\,\textrm{d}x. \end{aligned}$$

### Proof

Let $f\in L_{P}^{p}(\mathbb {R}^{d})$, $\psi \in {\mathcal {S}}_{P}$ and pick $\varphi$ so that $\psi =P\varphi$. By Hölder’s inequality we have

$$\begin{aligned} \left| \langle f,\psi \rangle _{{\mathcal {S}}'_{P},{\mathcal {S}}_{P}}\right| \le \int _{\mathbb {R}^{d}}\left| f(x)P(x)\varphi (x)\right| \,\textrm{d}x\le ||f||_{L_{P}^{p}}||\varphi ||_{L^{p'}}\le C||f||_{L_{P}^{p}}||\varphi ||_{k} \end{aligned}$$

for some $k\in \mathbb {N}$ and $C>0$ that depend only on *d* and *p*, where $p'$ is the conjugate exponent of *p*. By Thm. 1 of [4], the linear continuous multiplication map $M_{P}:{\mathcal {S}}\longrightarrow {\mathcal {S}}_{P}$ defined by $M_{P}\varphi =P\varphi$ has a continuous inverse $M_{P}^{-1}:{\mathcal {S}}_{P}\longrightarrow {\mathcal {S}}$. It follows that

$$\begin{aligned} \exists C'>0\,\exists k'\in \mathbb {N}\,\forall \psi \in {\mathcal {S}}_{P}:\,\left| \langle f,\psi \rangle _{{\mathcal {S}}'_{P},{\mathcal {S}}_{P}}\right| \le C'||f||_{L_{P}^{p}}||\psi ||_{k'} \end{aligned}$$

which completes the proof. $\square$

## The weak derivative in the sense of $L_{v,\textrm{loc}}^{1}(\Omega )$

Let $w\in E_{p}(\Omega )$. When *w* is arbitrary small, elements of $L_{w}^{p}(\Omega )$ are not necessary locally integrable over $\Omega$, and hence the notion of distributional derivative to elements of $L_{w}^{p}(\Omega )$ is not well defined in general. The existing remedy is to replace $\Omega$ with $\Omega _{|w|^{p}}^{*}:=\Omega \backslash P(|w|^{p})$ where $P(|w|^{p})$ is the set of points $x\in \Omega$ for which

$$\begin{aligned} |w|^{-p}\not \in L^{\frac{1}{p-1}}(\Omega \cap U) \end{aligned}$$

for all neighborhood *U* of *x*. The set $P(|w|^{p})$ is closed and Lebesgue negligible. Moreover, $L_{w}^{p}(\Omega )\subseteq L_{\text {loc}}^{1}(\Omega _{|w|^{p}}^{*})$ with continuous embedding (see Sect. 3 of [5] for more details). Hence, the notion of weak derivative in the sense of $L_{\textrm{loc}}^{1}(\Omega _{|w|^{p}}^{*})$ is well defined on the space $L_{w}^{p}(\Omega )$. However, note that with this weak derivative, we are allowed to take only test functions having a compact support in $\Omega _{|w|^{p}}^{*}$.

The main aim of this section is to present a new notion of weak derivative where the test functions are not necessary equal to 0 near the set $P(|w|^{p})$ as it is the case for the weak derivative in the sense of $L_{\textrm{loc}}^{1}(\Omega _{|w|^{p}}^{*})$.

### Definition 4

Let $\Omega \subseteq \mathbb {R}^{d}$ be an open set, $\alpha \in \mathbb {N}^{d}$ with $|\alpha |\ne 0$, $p\in [1,\infty )$, $w\in E_{p}(\Omega )$, and $v\in {\mathcal {C}}^{|\alpha |,*}(\Omega )$ (i.e. $v\in {\mathcal {C}}^{|\alpha |}(\Omega )$ and $v\ne 0$ a.e. in $\Omega$) be a map satisfying

3.1 $$\begin{aligned} \forall K\Subset \Omega \,\exists C>0:\,|v|\le C|w|\,\,\,\text {a.e. in }K. \end{aligned}$$

We say that $f\in L_{w,\text {loc}}^{1}(\Omega )$ is $\alpha$-weakly differentiable in the sense of $L_{v,\text {loc}}^{1}(\Omega )$ if there exists an element of $L_{v^{|\alpha |+1},\text {loc}}^{1}(\Omega )$ denoted by $D_{v}^{\alpha }f$ satisfying

3.2 $$\begin{aligned} \forall \varphi \in {\mathcal {D}}(\Omega ):\,\int _{\Omega }f\partial ^{\alpha }(v^{|\alpha |+1}\varphi )\,\textrm{d}x=(-1)^{|\alpha |}\int _{\Omega }v^{|\alpha |+1}\varphi D_{v}^{\alpha }f\,\textrm{d}x. \end{aligned}$$

In the sequel, for some given $f\in L_{w,\text {loc}}^{1}(\Omega )$, we will frequently write $\exists D_{v}^{\alpha }f$ to say that the $\alpha$-weak derivative of *f* in the sense of $L_{v,\text {loc}}^{1}(\Omega )$ exists (and hence belongs to $L_{v^{|\alpha |+1},\text {loc}}^{1}(\Omega )$).

### Remark 5

Let $\Omega$, $\alpha$, *p*, *w*, *v* be as in the previous definition.

(i) The weak derivative in the sense of $L_{v,\text {loc}}^{1}(\Omega )$ (when it exists) is unique. The proof is identical to the proof of the uniqueness of the weak derivative in the sense of $L_{\text {loc}}^{1}(\Omega )$.
(ii) Take $\alpha$ with $|\alpha |=1$ and let $f\in L_{w}^{p}(\Omega )$. Assume that $\exists D_{v}^{\alpha }f\in L_{v^{2}}^{p}(\Omega )$, and that $f\partial ^{\alpha }(v^{2})$, $v^{2}f\in L^{p}(\Omega )$, e.g. $\Omega$ is bounded and $\nabla v\in L^{\infty }(\Omega )$, then (3.2) yields
  $$\begin{aligned} \forall \varphi \in {\mathcal {D}}(\Omega ):\,\int _{\Omega }\left( f\partial ^{\alpha }(v^{2})+v^{2}D_{v}^{\alpha }f\right) \varphi \,\textrm{d}x=-\int _{\Omega }v^{2}f\partial ^{\alpha }\varphi \,\textrm{d}x \end{aligned}$$
  which implies that $v^{2}f$ is $\alpha$-weakly differentiable in the sense of $L_{\text {loc}}^{1}(\Omega )$ (or simply $\exists D^{\alpha }(v^{2}f)$) and $D^{\alpha }(v^{2}f)=f\partial ^{\alpha }(v^{2})+v^{2}D_{v}^{\alpha }f\in L^{p}(\Omega )$. Consequently, if we assume that the above conditions hold for all $\alpha \in \mathbb {N}^{d}$ with $|\alpha |=1$ we get that $v^{2}f\in W^{1,p}(\Omega )$.
(iii) One can show that for any $\varphi \in {\mathcal {D}}(\Omega )$, there exists a compactly supported continuous function $h_{\alpha }$ on $\Omega$ such that $\partial ^{\alpha }(v^{|\alpha |+1}\varphi )=vh_{\alpha }$. By (3.1) we have $|\partial ^{\alpha }(v^{|\alpha |+1}\varphi )|\le C|w|h_{\alpha }$ a.e. on $K:=\text {supp}(\varphi )$. It follows that
  $$\begin{aligned} \left| \int _{\Omega }f\partial ^{\alpha }(v^{|\alpha |+1}\varphi )\,\textrm{d}x\right| \le C||f||_{L_{w}^{p}(\Omega )}||h_{\alpha }||_{L^{\infty }(K)}. \end{aligned}$$
  Hence, the left hand side of (3.2) is always finite. Moreover, for any test function $\varphi$, the functional $L_{w}^{p}(\Omega )\longrightarrow {\mathbb {C}}$, $f\longrightarrow \int _{\Omega }f\partial ^{\alpha }(v^{|\alpha |+1}\varphi )\,\textrm{d}x$ is continuous. This property will be used to prove the completeness of the spaces $W_{V,v}^{m,p}(\Omega )$ defined below.
(iv) By density of ${\mathcal {D}}(\Omega )$ in ${\mathcal {C}}_{c}^{|\alpha |}(\Omega )$, we can equally well use the space ${\mathcal {C}}_{c}^{|\alpha |}(\Omega )$ instead of the space ${\mathcal {D}}(\Omega )$ in (3.2). This is because using (3.1) we have
  $$\begin{aligned} \left| \int _{\Omega }f\partial ^{\alpha }(v^{|\alpha |+1}\varphi )\,\textrm{d}x\right| \le C||fw||_{L^{1}(K)}\sum _{0\le \beta \le \alpha }C_{\beta }||g_{\beta }||_{L^{\infty }(K)}||\partial ^{\beta }\varphi ||_{L^{\infty }(K)}, \end{aligned}$$
  where *K* is the support of $\varphi$, $g_{\beta }$ is a continuous function that depends on *v*, $\alpha$ and $\beta$.
(v) In case $w\in {\mathcal {C}}^{|\alpha |}(\Omega )$ we can simply choose $v=w$.
(vi) The case $w\equiv 1$ can be considered since (3.1) trivially holds.

### Example 6

Let $$f(x)=1/x^{2}\in L_{x^{\text {2}}}^{2}(-1,1)$$. Then, $$\exists D_{x^{2}}f=-2x^{-3}\in L_{x^{3}}^{2}(-1,1)$$.

Now, we compare the notion of weak derivative in the sense of $L_{v,\textrm{loc}}^{1}(\Omega )$ with the notion of the weak derivative in the sense of $L_{\textrm{loc}}^{1}$.

If *m*, $k\in \mathbb {N}$ are such that $k<m$, then $\pi _{m}^{k}:=\pi _{m}\backslash \pi _{k}$. More generally, if $\alpha \in \mathbb {N}^{d}$, then $\pi _{\alpha }$ is the set of multi-indices $\beta \in \mathbb {N}^{d}$ such that $\beta \le \alpha$ (i.e. $\beta _{i}\le \alpha _{i}$ for all *i*). If $\lambda \in \mathbb {N}^{d}$ is such that $\lambda <\alpha$ (i.e. $\lambda \le \alpha$ with $\lambda _{j}<\alpha _{j}$ for some *j*), then $\pi _{\alpha }^{\lambda }$ denotes the set $\pi _{\alpha }\backslash \pi _{\lambda }$.

The relation between the week derivative in the sense of $L_{\textrm{loc}}^{1}$ and the weak derivative in the sense of $L_{v,\textrm{loc}}^{1}(\Omega )$ is given in the next proposition.

### Proposition 7

Let $\Omega \subseteq \mathbb {R}^{d}$ be an open set, $\alpha \in \mathbb {N}^{d}$ with $|\alpha |\ne 0$, $p\in [1,\infty )$, $w\in E_{p}(\Omega )$, $v\in {\mathcal {C}}^{|\alpha |,*}(\Omega )$ be a map satisfying (3.1).

(i) Let $f\in L_{\textrm{loc}}^{1}(\Omega )$($=L_{w,\textrm{loc}}^{1}(\Omega )$ with $w\equiv 1$). If $\exists D^{\alpha }f\in L_{\textrm{loc}}^{1}(\Omega )$ then $\exists D_{v}^{\alpha }f$ and $D^{\alpha }f=D_{v}^{\alpha }f$.
(ii) Let $f\in L_{w,\textrm{loc}}^{p}(\Omega )$. Then, *f* is $\alpha$-weakly differentiable in the sense of $L_{v,\textrm{loc}}^{1}(\Omega )$ with $D_{v}^{\alpha }f\in L_{v^{|\alpha |+1},\textrm{loc}}^{p}(\Omega )$ if and only if its $\alpha$-weak derivative in the sense of $L_{\textrm{loc}}^{1}(\Omega _{|v|^{p|\alpha |+p}}^{*})$, denoted by $D^{\alpha }f$, exists and belongs to $L_{v^{|\alpha |+1},\textrm{loc}}^{p}(\Omega )$. In particular, we have $D_{v}^{\alpha }f=D^{\alpha }f$ a.e. in $\Omega _{|v|^{p|\alpha |+p}}^{*}$.

### Proof

(i): Recall that, by density of ${\mathcal {D}}(\Omega )$ in the space ${\mathcal {C}}_{c}^{|\alpha |}(\Omega )$, we can replace the space ${\mathcal {D}}(\Omega )$ in the definition of the weak derivative in the sense of $L_{\text {loc}}^{1}(\Omega )$ with the space ${\mathcal {C}}_{c}^{|\alpha |}(\Omega )$. Hence, it suffices to use the definition of the weak derivative in the sense of $L_{\text {loc}}^{1}(\Omega )$ with the test function $\varphi v^{|\alpha |+1}\in {\mathcal {C}}_{c}^{|\alpha |}(\Omega )$ instead of $\varphi \in {\mathcal {D}}(\Omega )$.

(ii): Assume that *f* is $\alpha$-weakly differentiable in the sense of $L_{v,\textrm{loc}}^{1}(\Omega )$ with $D_{v}^{\alpha }f\in L_{v^{|\alpha |+1},\textrm{loc}}^{p}(\Omega )$. Let $\eta \in {\mathcal {C}}^{\infty }(\mathbb {R})$ be such that

$$\begin{aligned} \eta \equiv 1\,\,\,\text {on }\,\left[ -1,1\right] ^{c},\,\eta \equiv 0\text { on }\left[ -\frac{1}{2},\frac{1}{2}\right] ,\,0\le \eta \le 1, \end{aligned}$$

and for all $n\in \mathbb {N}$, set $\chi _{n}:=\eta _{n}\circ v$, where $\eta _{n}(\cdot ):=\eta (n\cdot )$. Setting

$$\begin{aligned} \forall n\in \mathbb {N}:\,M_{n}:=\left\{ x\in \Omega \mid |v(x)|\le 1/n\right\} \end{aligned}$$

Clearly $$\chi _{n}\in {\mathcal {C}}^{|\alpha |}(\Omega )$$, and

3.3 $$\begin{aligned} \forall n\in \mathbb {N}:\,\chi _{n}\equiv 1\,\,\,\text {on }M_{n}^{c},\,\chi _{n}\equiv 0\text { on }M_{2n},\,\text {and }0\le \chi _{n}\le 1 \end{aligned}$$

where $M_{n}^{c}$ is the complementary (within $\Omega$) of $M_{n}$. For any $\varphi \in {\mathcal {D}}(\Omega _{|v|^{p|\alpha |+p}}^{*})$, we are allowed to use (3.2) with $\chi _{n}\varphi v^{-|\alpha |-1}\in {\mathcal {C}}_{c}^{|\alpha |}(\Omega _{|v|^{p|\alpha |+p}}^{*})$ (see (iv) of Remark 5). Thus, we obtain

$$\begin{aligned} \sum _{0\le \beta \le \alpha }{\alpha \atopwithdelims ()\beta }\int _{\Omega }f\partial ^{\beta }\chi _{n}\partial ^{\alpha -\beta }\varphi \,\textrm{d}x=(-1)^{|\alpha |}\int _{\Omega }\chi _{n}\varphi D_{v}^{\alpha }f\,\textrm{d}x. \end{aligned}$$

By the continuous inclusions $L_{w,\textrm{loc}}^{p}(\Omega )\subseteq L_{v,\textrm{loc}}^{p}(\Omega )\subseteq L_{v^{|\alpha |+1},\textrm{loc}}^{p}(\Omega )\subseteq L_{\textrm{loc}}^{1}(\Omega _{v^{p|\alpha |+p}}^{*})$ (the first inclusion follows from (3.1), and the latter inclusion follows from Thm. 1.5 of [5]) *f*, $D_{v}^{\alpha }f\in L_{\textrm{loc}}^{1}(\Omega _{|v|^{p|\alpha |+p}}^{*})$. Hence, by the dominated convergence theorem, we have that

$$\begin{aligned} \int _{\Omega }\chi _{n}\varphi D_{v}^{\alpha }f\,\textrm{d}x.\longrightarrow \int _{\Omega }\varphi D_{v}^{\alpha }f\,\textrm{d}x\,\,\,\text {and}\,\,\,\int _{\Omega }f\chi _{n}\partial ^{\alpha }\varphi \,\textrm{d}x\longrightarrow \int _{\Omega }f\partial ^{\alpha }\varphi \,\textrm{d}x. \end{aligned}$$

Left to show that

3.4 $$\begin{aligned} \forall \beta \in \pi _{\alpha }^{0}:\,\int _{\Omega }f\partial ^{\beta }(\chi _{n})\partial ^{\alpha -\beta }\varphi \,\textrm{d}x\longrightarrow 0. \end{aligned}$$

Fix $\beta \in \pi _{\alpha }^{0}$. Note that $\partial ^{\beta }\chi _{n}\rightarrow 0$ pointwise a.e. in $\Omega$. On the other hand, the Faà di Bruno formula ( [7]) gives

$$\begin{aligned} \partial ^{\beta }\chi _{n}(x)=\sum _{k=1}^{|\beta |}n^{k}|\{\pi \in \Pi \,\big |\,|\pi |=k\}|\eta ^{(k)}(nv(x))\sum _{\pi \in \Pi ,|\pi |=k}\prod _{B\in \pi }\frac{\partial ^{|B|}v}{\prod _{j\in B}\partial x_{j}} \end{aligned}$$

where

- $\pi$ runs through the set $\Pi$ of all partitions of the set ${\mathcal {A}}$ of non-null indices of $\beta$ with multiplicity e.g. if $\beta =(2,3,0,1)$ then ${\mathcal {A}}:=\{1,1,2,2,2,4\}$
- “$B\in \pi$” means the variable *B* runs through the list of all of the “blocks” of the partition $\pi$, and
- |*A*| denotes the cardinality of the set *A* (so that $|\pi |$ is the number of blocks in the partition $\pi$ and |*B*| is the size of the block *B*).

By construction, $\partial ^{\beta }\chi _{n}(x)=0$ for all $x\in M_{n}^{c}\cup M_{2n}$. Let now $x\in M_{n}\cap M_{2n}^{c}$. Since $v\in {\mathcal {C}}^{|\alpha |}(\Omega )$, we have that

$$\begin{aligned} \exists C>0\,\forall n\in \mathbb {N}\,\forall x\in \text {supp}(\varphi )\cap M_{n}\cap M_{2n}^{c}:\,|\partial ^{\beta }\chi _{n}(x)|\le Cn^{|\beta |}, \end{aligned}$$

and hence

$$\begin{aligned} \exists C>0\,\forall n\in \mathbb {N}\,\forall x\in \text {supp}(\varphi )\cap M_{n}\cap M_{2n}^{c}:\,|v^{|\alpha |}\partial ^{\beta }\chi _{n}(x)|\le Cn^{|\beta |-|\alpha |}\le C. \end{aligned}$$

It follows that

$$\begin{aligned} \exists C>0\,\forall n\in \mathbb {N}:\,\left| f\partial ^{\beta }(\chi _{n})\partial ^{\alpha -\beta }\varphi \right| \le C\left| v^{-|\alpha |}f\partial ^{\alpha -\beta }\varphi \right| \,\,\,\text {a.e. in }\Omega . \end{aligned}$$

Since $f\in L_{v,\textrm{loc}}^{p}(\Omega )$ (by (3.1)) and $v^{-|\alpha |-1}\in L_{\textrm{loc}}^{\frac{p}{p-1}}(\Omega _{|v|^{p|\alpha |+p}}^{*})$ (which follows from the definition of the set $\Omega _{|v|^{p|\alpha |+p}}^{*}$), Hölder’s inequality implies that $v^{-|\alpha |}f\partial ^{\alpha -\beta }\varphi \in L^{1}(\Omega )$. Therefore, (3.4) is now a consequence of the dominated convergence theorem.

Assume now that *f* is $\alpha$-weakly differentiable in the sense of $L_{\textrm{loc}}^{1}(\Omega _{|v|^{p|\alpha |+p}}^{*})$ with $D^{\alpha }f\in L_{v^{|\alpha |+1},\textrm{loc}}^{p}(\Omega )$. Let $\chi _{n}$ be defined as above. Since $\chi _{n}v^{|\alpha |+1}\varphi \in {\mathcal {C}}_{c}^{|\alpha |}(\Omega _{v^{p|\alpha |+p}}^{*})$, we have

3.5 $$\begin{aligned}  &   \sum _{0\le \beta \le \alpha }{\alpha \atopwithdelims ()\beta }\int _{\Omega }f\partial ^{\beta }(\chi _{n})\partial ^{\alpha -\beta }(v^{|\alpha |+1}\varphi )\,\textrm{d}x\nonumber \\  &   \quad =\int _{\Omega }f\partial ^{\alpha }(v^{|\alpha |+1}\chi _{n}\varphi )\,\textrm{d}x=(-1)^{|\alpha |}\int _{\Omega }v^{|\alpha |+1}\chi _{n}\varphi D^{\alpha }f\,\textrm{d}x. \end{aligned}$$

One can easily show by induction that

3.6 $$\begin{aligned} \forall \beta \in \pi _{\alpha }\,\exists g_{\beta }\in {\mathcal {C}}_{c}^{|\beta |}(\Omega ):\,\partial ^{\alpha -\beta }(v^{|\alpha |+1}\varphi )=v^{|\beta |+1}g_{\beta }. \end{aligned}$$

As shown above, it is easy to see that

$$\begin{aligned} \forall \beta \in \pi _{\alpha }\,\exists C>0\,\forall n\in \mathbb {N}:\,\left| f\partial ^{\alpha -\beta }(v^{|\alpha |+1}\varphi )\partial ^{\beta }\chi _{n}\right| \le C|fvg_{\beta }| \end{aligned}$$

By the dominated convergence theorem (taking the limit in (3.5)) we get

$$\begin{aligned} \int _{\Omega }f\partial ^{\alpha }(v^{|\alpha |+1}\varphi )\,\textrm{d}x=(-1)^{|\alpha |}\int _{\Omega }v^{|\alpha |+1}\varphi D^{\alpha }f\,\textrm{d}x, \end{aligned}$$

which completes the proof. $\square$

## The weighted Sobolev space $W_{V,v}^{m,p}(\Omega )$

If $V:=\{w_{\alpha }\}_{\alpha \in \pi _{m}}$ is a collection of elements of $E_{p}(\Omega )$, then the function $w_{0}$ ($0\in \mathbb {N}^{d}$) will be simply denoted by *w*.

### Definition 8

Let $\Omega \subseteq \mathbb {R}^{d}$ be an open set, $p\in [1,\infty )$, $m\in \mathbb {N}$, and $V:=\{w_{\alpha }\}_{\alpha \in \pi _{m}}$ be a collection of elements of $E_{p}(\Omega )$, $v\in {\mathcal {C}}^{m,*}(\Omega )$ be a map satisfying (3.1). We set

$$\begin{aligned} W_{V,v}^{m,p}(\Omega ):=\{f\in L_{w}^{p}(\Omega )\mid \forall \alpha \in \pi _{m}^{0}:\,\exists D_{v}^{\alpha }f\in L_{w_{\alpha }}^{p}(\Omega )\}. \end{aligned}$$

This space is equipped with the norm

$$\begin{aligned} \forall f\in W_{V,v}^{m,p}(\Omega ):\,||f||_{W_{V,v}^{m,p}}:=\left( \sum _{|\alpha |\le m}||D_{v}^{\alpha }f||_{L_{w_{\alpha }}^{p}}^{p}\right) ^{1/p}. \end{aligned}$$

When $p=2$, the space $W_{V,v}^{m,2}(\Omega )$ will be denoted by $H_{V,v}^{m}(\Omega )$ and its norm is generated by the scalar product

$$\begin{aligned} \forall f,g\in H_{V,v}^{m}(\Omega ):\,(f,g)_{H_{V,v}^{m}}:=\sum _{|\alpha |\le m}(D_{v}^{\alpha }f,D_{v}^{\alpha }g)_{L_{w_{\alpha }}^{2}}. \end{aligned}$$

We now prove that the space $W_{V,v}^{m,p}(\Omega )$ is Cauchy complete.

### Theorem 9

Let $\Omega \subseteq \mathbb {R}^{d}$ be an open set, $p\in [1,\infty )$, $m\in \mathbb {N}$, and let $V:=\{w_{\alpha }\}_{\alpha \in \pi _{m}}$ be a collection of elements of $E_{p}(\Omega )$, and $v\in {\mathcal {C}}^{m,*}(\Omega )$. Assume that

4.1 $$\begin{aligned} \forall \alpha \in \pi _{m}\,\forall K\Subset \Omega \,\exists C_{K}:\,|v(x)|^{|\alpha |+1}\le C_{K}|w_{\alpha }(x)|\,\,\,\text {a.e. in }K. \end{aligned}$$

Then, the space $W_{V,v}^{m,p}(\Omega )$ is Cauchy complete.

### Proof

Let $(f_{n})$ be a Cauchy sequence of $W_{V,v}^{m,p}(\Omega )$. By definition, for all $\alpha \in \pi _{m}$, the sequence $(D_{v}^{\alpha }f_{n})$ is a Cauchy sequence of $L_{w_{\alpha }}^{p}(\Omega )$. By completeness of $L_{w_{\alpha }}^{p}(\Omega )$ (see Theorem 2) there exists a unique $f_{\alpha }\in L_{w_{\alpha }}^{p}(\Omega )$ limit of the sequence $(D_{v}^{\alpha }f_{n})$ in $L_{w_{\alpha }}^{p}(\Omega )$. Set $f:=f_{0}$. We shall prove that *f* is weakly differentiable in the sense of $L_{v,\text {loc}}^{1}(\Omega )$ with $D_{v}^{\alpha }f=f_{\alpha }$ for all $\alpha \in \pi _{m}^{0}$. Indeed, by assumption we have

4.2 $$\begin{aligned} \forall \varphi \in {\mathcal {D}}(\Omega ):\,\int _{\Omega }f_{n}\partial ^{\alpha }\left( v^{|\alpha |+1}\varphi \right) \,\textrm{d}x=(-1)^{|\alpha |}\int _{\Omega }\varphi v^{|\alpha |+1}D_{v}^{\alpha }f_{n}\,\textrm{d}x. \end{aligned}$$

We have $\lim _{n\rightarrow \infty }\int _{\Omega }f_{n}\partial ^{\alpha }\left( v^{|\alpha |+1}\varphi \right) \,\textrm{d}x=\int _{\Omega }f\partial ^{\alpha }\left( v^{|\alpha |+1}\varphi \right) \,\textrm{d}x$ by (iii) of Remark 5. On the other hand, (4.1) implies that

$$\begin{aligned} \left| \int _{\Omega }(D_{v}^{\alpha }f_{n})v^{|\alpha |+1}\varphi \,\textrm{d}x\right| \le C_{\text {supp}(\varphi )}\int _{\Omega }\left| (D_{v}^{\alpha }f_{n})w_{\alpha }\varphi \right| \,\textrm{d}x\le C_{\text {supp}(\varphi )}||D_{v}^{\alpha }f_{n}||_{L_{w_{\alpha }}^{p}}||\varphi ||_{L^{q}} \end{aligned}$$

where *q* is the conjugate exponent of *p*, which implies that the right hand side of (4.2) converges to $(-1)^{|\alpha |}\int _{\Omega }\varphi v^{|\alpha |+1}f_{\alpha }\,\textrm{d}x$ when $n\rightarrow \infty$. It follows that

$$\begin{aligned} \forall \varphi \in {\mathcal {D}}(\Omega ):\,\int _{\Omega }f\partial ^{\alpha }\left( v^{|\alpha |+1}\varphi \right) \,\textrm{d}x=(-1)^{|\alpha |}\int _{\Omega }\varphi v^{|\alpha |+1}f_{\alpha }\,\textrm{d}x \end{aligned}$$

which proves that $\exists D_{v}^{\alpha }f=f_{\alpha }\in L_{w_{\alpha }}^{p}(\Omega )\subseteq L_{v^{|\alpha |+1},\text {loc}}^{1}(\Omega )$ (where the last inclusion follows from (4.1)), and hence $f\in W_{V,v}^{m,p}(\Omega )$. Therefore, the sequence $(f_{n})$ converges to *f* in $W_{V,v}^{m,p}(\Omega )$, and the proof is completed. $\square$

### Remark 10

If $w_{\alpha }=|v|^{\sigma }$ for some $\sigma \in (0,|\alpha |+1)$ then (4.1) holds. This is because $|v|^{|\alpha |+1-\sigma }$ is bounded on any compact subset of $\Omega$.

We now compare the new approach of weighted Sobolev spaces presented in this paper with the classical one.

Let $V:=\{w_{\alpha }\}_{\alpha \in \pi _{m}}$ be a collection of elements of $E_{p}(\Omega )$, and let $S:=\{u_{\alpha }\}_{\alpha \in \pi _{m}}$ where $u_{\alpha }=|w_{\alpha }|^{p}\in W(\Omega )$. We recall that the weighted Sobolev space $W^{m,p}(\Omega ,S)$ is defined as the space of all functions $f\in L^{p}(\Omega ,u)\cap L_{\textrm{loc}}^{1}(\Omega )$ such that their distributional derivative are elements of $L^{p}(\Omega ,u_{\alpha })\cap L_{\textrm{loc}}^{1}(\Omega )$. The space $W^{m,p}(\Omega ,S)$ is equipped with the norm

$$\begin{aligned} \forall f\in W^{m,p}(\Omega ,S):\,||f||_{W^{m,p}(\Omega ,S)}:=\left( \sum _{|\alpha |\le m}||u_{\alpha }^{1/p}D^{\alpha }f||_{L^{p}}^{p}\right) ^{1/p}. \end{aligned}$$

In order to guarantee the Cauchy completeness of $W^{m,p}(\Omega ,S)$ we assume that

4.3 $$\begin{aligned} \forall \alpha \in \pi _{m}:\,u_{\alpha }^{-1}\in L_{\textrm{loc}}^{\frac{1}{p-1}}(\Omega ) \end{aligned}$$

(see Thm. 1.11 of [5]). However, when (4.3) is violated, the weighted Sobolev space $W^{m,p}(\Omega ,S)$ is not necessarily a Banach space (see Example 1.12 of [5]). Indeed, the “bad” set which may cause the non-completeness of $W^{m,p}(\Omega ,S)$ is the set $B:=\cup _{\alpha \in \pi _{m}}P(u_{\alpha })$ where $P(u_{\alpha })$ is the set of points $x\in \Omega$ for which $u_{\alpha }^{-1}\not \in L^{\frac{1}{p-1}}(\Omega \cap U)$ for all neighborhood *U* of *x*. The existing remedy is to define the space $W^{m,p}(\Omega ,S)$ as the space $W^{m,p}(\Omega _{S}^{*},S)$ where $\Omega _{S}^{*}:=\Omega \backslash B$. It is shown that the set *B* is closed (and hence $\Omega _{S}^{*}$ is open) and Lebesgue negligible. Moreover, assumption (4.3) holds if we replace $\Omega$ with $\Omega _{S}^{*}$. Therefore, the weighted Sobolev space $W^{m,p}(\Omega ,S)$($=W^{m,p}(\Omega _{S}^{*},S)$) is now Cauchy complete (see Sect. 3 of [5] for more details). However, the space of test functions used in the definition of the distributional derivative is, in general, limited to the space ${\mathcal {C}}_{c}^{m}(\Omega _{S}^{*})$.

On the other hand, if there exists a ${\mathcal {C}}^{m,*}(\Omega )$ function *v* that satisfies (4.1), then, by assertion (ii) of Proposition 7, we have that

4.4 $$\begin{aligned} W^{m,p}(\Omega _{S}^{*},S)\subseteq W^{m,p}(\Omega _{|v|^{pm+p}}^{*},S)=W_{V,v}^{m,p}(\Omega ). \end{aligned}$$

In case $\Omega _{S}^{*}=\Omega _{v^{pm+p}}^{*}$ e.g. $w_{\alpha }:=v^{|\alpha |+1}$, the inclusion in (4.4) becomes an equality.

In conclusion, we have the following remark

### Remark 11

For a given collection $S:=\{|w_{\alpha }|^{p}\}_{\alpha \in \pi _{m}}$ of arbitrarily small weight functions, the weighted Sobolev space $W^{m,p}(\Omega _{S}^{*},S)$ is a Banach space while the space $W^{m,p}(\Omega ,S)$ in general is not. The price we pay is a modification of the domain and hence the convexity property might be lost and the boundary of $\Omega$ is modified. If there exists a ${\mathcal {C}}^{m,*}(\Omega )$ function *v* satisfying (4.1), then we can consider the space $W_{V,v}^{m,p}(\Omega )$ (where $V:=\{w_{\alpha }\}_{\alpha \in \pi _{m}}$) instead of $W^{m,p}(\Omega _{S}^{*},S)$, which is in general larger. In this case, no modification of the domain is needed and elements of $W_{V,v}^{m,p}(\Omega )$ are weakly differentiable in the sense of $L_{\textrm{loc}}^{1}(\Omega _{|v|^{mp+p}}^{*})$ and in the sense of $L_{v,\textrm{loc}}^{1}(\Omega )$ but not necessary in the sense of $L_{\textrm{loc}}^{1}(\Omega _{S}^{*})$. If furthermore, the function *v* is well chosen so that $\Omega _{S}^{*}=\Omega _{|v|^{pm+p}}^{*}$, then $W^{m,p}(\Omega _{S}^{*},S)=W_{V,v}^{m,p}(\Omega ).$

Let $m\in \mathbb {N}$ and let $s:\pi _{m}\longrightarrow \mathbb {R}_{\ge 0}$ be a map having the following properties

4.5 $$\begin{aligned} \forall \alpha \in \pi _{m}\,\forall \beta \in \pi _{\alpha }:\,s(\alpha )\le |\alpha |,\,\,\,\text {and}\,\,\,s(\alpha -\beta )+s(\beta )\le s(\alpha ) \end{aligned}$$

A typical example of *s* is the map $|\cdot |$. Let $v\in {\mathcal {C}}^{m,*}(\Omega )$, and consider the collection $V=\{w_{\alpha }\}_{\alpha \in \pi _{m}}$ of elements of $E_{p}(\Omega )$ defined by

$$\begin{aligned} \forall \alpha \in \pi _{m}:w_{\alpha }:=|v|^{s(\alpha )+1}. \end{aligned}$$

Then, clearly (4.1) holds since $|\alpha |-s(\alpha )\ge 0$. It follows that the space $W_{s,v}^{m,p}(\Omega ):=W_{V,v}^{m,p}(\Omega )$ is a Banach space (see Theorem 9).

### Proposition 12

Let $\Omega \subseteq \mathbb {R}^{d}$ be an open set, $p\in [1,\infty )$, $m\in \mathbb {N}$, $v\in {\mathcal {C}}^{m,*}(\Omega )$, $s:\pi _{m}\longrightarrow \mathbb {R}_{\ge 0}$ be a map satisfying (4.5). Then, the operator $W_{s,v}^{m,p}(\Omega )\longrightarrow W_{\textrm{loc}}^{m,p}(\Omega )$, $f\longrightarrow v^{m+1}f$ is continuous. Moreover

4.6 $$\begin{aligned} \forall f\in W_{s,v}^{m,p}(\Omega )\,\forall \alpha \in \pi _{m}:\,D^{\alpha }(v^{m+1}f)=\sum _{0\le \beta \le \alpha }{\alpha \atopwithdelims ()\beta }D_{v}^{\beta }f\partial ^{\alpha -\beta }v^{m+1}. \end{aligned}$$

In particular, one can replace $W_{\textrm{loc}}^{m,p}(\Omega )$ with $W^{m,p}(\Omega )$ in case $v\in {\mathcal {C}}_{b}^{m}(\Omega )$.

### Proof

Let $f\in W_{s,v}^{m,p}(\Omega )$ and $\alpha \in \pi _{m}$. For any $\sigma \in \pi _{\alpha }$, there exists $g_{\sigma }\in {\mathcal {C}}^{m-|\sigma |}(\Omega )$ such that $\partial ^{\sigma }v^{m+1}=g_{\sigma }v^{m+1-|\sigma |}$, which implies that

4.7 $$\begin{aligned} \forall K\Subset \Omega :\!\left\| D_{v}^{\beta }f\partial ^{\alpha -\beta }v^{m+1}\right\| _{L^{p}(K)}{\le } C||v^{m-|\alpha |}g_{\alpha -\beta }||_{L^{\infty }(K)}\left\| |v|^{s(\beta )+1}D_{v}^{\beta }f\right\| _{L^{p}(\Omega )}\nonumber \\ \end{aligned}$$

where we used (4.1) (with $w_{\beta }=|v|^{s(\beta )+1}$) in the last inequality, which shows that the right hand side of (4.6) belongs to $L_{\textrm{loc}}^{p}(\Omega )$ for all $\alpha \in \pi _{m}$. In case $v\in {\mathcal {C}}_{b}^{m}(\Omega )$, $v^{m-|\alpha |}g_{\alpha -\beta }\in L^{\infty }(\Omega )$ and the constant *C* does not depend on the compact set *K*. Thus, (4.7) holds with $\Omega$ instead of *K*. Therefore, the right hand side of (4.6) belongs to $L^{p}(\Omega )$ for all $\alpha \in \pi _{m}$. We show now that $v^{m+1}f$ is weakly differentiable in the sense of $L_{\textrm{loc}}^{1}(\Omega )$ and that the Leibniz formula (4.6) holds. Indeed, we will prove this by induction. Suppose first that $\alpha \in \pi _{1}^{0}$. Choose $\varphi \in {\mathcal {D}}(\Omega )$. We have

$$\begin{aligned} \int _{\Omega }fv^{m+1}\partial ^{\alpha }\varphi \,\textrm{d}x= &   \int _{\Omega }f(\partial ^{\alpha }(v^{m+1}\varphi )-\varphi \partial ^{\alpha }v^{m+1})\,\textrm{d}x\\= &   -\int _{\Omega }(v^{m+1}D_{v}^{\alpha }f+f\partial ^{\alpha }v^{m+1})\varphi \,\textrm{d}x, \end{aligned}$$

where we used (3.2) with the test function $\varphi v^{m-|\alpha |}\in {\mathcal {C}}_{c}^{m}(\Omega )$, which proves (4.6) when $\alpha \in \pi _{1}^{0}$. We assume now that (4.6) holds for all $\alpha \in \pi _{l}^{0}$ for some $l<m$. Choose now $\alpha \in \pi _{l+1}^{l}$. Then $\alpha =\beta +\gamma$ for some $|\beta |=l$ and $|\gamma |=1$. Then for $\varphi$ as above,

$$\begin{aligned} \int _{\Omega }fv^{m+1}\partial ^{\alpha }\varphi \,\textrm{d}x= &   (-1)^{|\beta |}\int _{\Omega }D^{\beta }(fv^{m+1})\partial ^{\gamma }\varphi \,\textrm{d}x\\= &   (-1)^{|\beta |}\sum _{0\le \sigma \le \beta }{\beta \atopwithdelims ()\sigma }\int _{\Omega }D_{v}^{\sigma }f\partial ^{\beta -\sigma }v^{m+1}\partial ^{\gamma }\varphi \,\textrm{d}x \end{aligned}$$

where we used the induction assumption. Using the equality

$$\begin{aligned} \partial ^{\beta -\sigma }v^{m+1}\partial ^{\gamma }\varphi =\partial ^{\gamma }(\varphi \partial ^{\beta -\sigma }v^{m+1})-\varphi \partial ^{\alpha -\sigma }v^{m+1} \end{aligned}$$

we get

$$\begin{aligned} \int _{\Omega }fv^{m+1}\partial ^{\alpha }\varphi \,\textrm{d}x= &   (-1)^{|\beta |}\sum _{0\le \sigma \le \beta }{\beta \atopwithdelims ()\sigma }\int _{\Omega }D_{v}^{\sigma }f\partial ^{\gamma }(\varphi \partial ^{\beta -\sigma }v^{m+1})\,\textrm{d}x\\  &   +(-1)^{|\alpha |}\sum _{0\le \sigma \le \beta }{\beta \atopwithdelims ()\sigma }\int _{\Omega }\varphi D_{v}^{\sigma }f\partial ^{\alpha -\sigma }v^{m+1}\,\textrm{d}x. \end{aligned}$$

Again, using (3.2) we obtain

$$\begin{aligned} \int _{\Omega }D_{v}^{\sigma }f\partial ^{\gamma }(\varphi \partial ^{\beta -\sigma }v^{m+1})\,\textrm{d}x=-\int _{\Omega }\varphi \partial ^{\beta -\sigma }v^{m+1}D_{v}^{\gamma +\sigma }f\,\textrm{d}x \end{aligned}$$

Thus

$$\begin{aligned} \int _{\Omega }fv^{m+1}\partial ^{\alpha }\varphi \,\textrm{d}x=(-1)^{|\alpha |}\sum _{0\le \sigma \le \beta }{\beta \atopwithdelims ()\sigma }\int _{\Omega }\varphi (D_{v}^{\rho }f\partial ^{\alpha -\rho }v^{m+1}+D_{v}^{\sigma }f\partial ^{\alpha -\sigma }v^{m+1})\,\textrm{d}x, \end{aligned}$$

where $\rho =\sigma +\gamma$. It follows that

$$\begin{aligned} \int _{\Omega }fv^{m+1}\partial ^{\alpha }\varphi \,\textrm{d}x=(-1)^{|\alpha |}\sum _{0\le \sigma \le \alpha }{\alpha \atopwithdelims ()\sigma }\int _{\Omega }\varphi (D_{v}^{\sigma }f\partial ^{\alpha -\sigma }v^{m+1})\,\textrm{d}x, \end{aligned}$$

since

$$\begin{aligned} {\beta \atopwithdelims ()\sigma -\gamma }+{\beta \atopwithdelims ()\sigma }={\alpha \atopwithdelims ()\sigma }, \end{aligned}$$

Therefore, the map $W_{s,v}^{m,p}(\Omega )\longrightarrow W_{\textrm{loc}}^{m,p}(\Omega )$, $f\longrightarrow v^{m+1}f$ is well defined and (4.6) holds. Moreover, the continuity follows from (4.6) and (4.7), which completes the proof. $\square$

## Density of smooth functions

In this section, we are interested in finding sufficient conditions under which smooth functions are dense in weighted Sobolev spaces $W_{s,v}^{m,p}(\Omega )$.

Let $v\in {\mathcal {C}}^{m,*}(\Omega )$, and let $s:\pi _{m}\longrightarrow \mathbb {R}_{\ge 0}$ be a map satisfying (4.5). In order to prove the density of smooth functions in $W_{s,v}^{m,p}(\Omega )$, we show first that

5.1 $$\begin{aligned} \forall f\in W_{s,v}^{m,p}(\Omega )\,\forall n\in \mathbb {N}:\,f\chi _{n}\in W^{m,p}(\Omega ), \end{aligned}$$

where $(\chi _{n})$ is the sequence constructed in Proposition 7. After that, we show that the sequence $(f\chi _{n})$ converges to *f* in $W_{s,v}^{m,p}(\Omega )$. Then, we use the density results of smooth functions in Sobolev spaces $W^{m,p}(\Omega )$ to conclude.

### Theorem 13

Let $\Omega \subseteq \mathbb {R}^{d}$ be an open set, $p\in [1,\infty )$, $m\in \mathbb {N}$, $v\in {\mathcal {C}}^{m,*}(\Omega )$, $s:\pi _{m}\longrightarrow \mathbb {R}_{\ge 0}$ be a map satisfying (4.5). Assume that

5.2 $$\begin{aligned} \forall \alpha \in \pi _{m}^{0}\,\exists C>0\,\forall x\in M_{2n}^{c}\cap M_{n}:\,|\partial ^{\alpha }v(x)|\le C. \end{aligned}$$

Then, the mapping $W_{s,v}^{m,p}(\Omega )\longrightarrow W^{m,p}(\Omega )$, $f\longrightarrow \chi _{n}f$ is continuous. In particular, for any $f\in W_{s,v}^{m,p}(\Omega )$ and for all $\alpha \in \pi _{m}^{0}$ we have

5.3 $$\begin{aligned} D^{\alpha }(f\chi _{n})=\sum _{0\le \beta \le \alpha }{\alpha \atopwithdelims ()\beta }D_{v}^{\beta }f\partial ^{\alpha -\beta }\chi _{n}. \end{aligned}$$

### Proof

First note that $\chi _{n}\in {\mathcal {C}}_{b}^{m}(\Omega )$ i.e. $\partial ^{\alpha }\chi _{n}$ is bounded on $\Omega$ for all $\alpha \in \pi _{m}$. This is because $\eta _{n}^{(k)}\equiv 0$ on $M_{2n}\cup M_{n}^{c}$ for all $k\ne 0$, and $\partial ^{\alpha }v$ is bounded on $M_{2n}^{c}\cap M_{n}$. Let $f\in W_{s,v}^{m,p}(\Omega )$. We show now that $D_{v}^{\beta }f\partial ^{\alpha -\beta }\chi _{n}\in L^{p}(\Omega )$ for all $\alpha \in \pi _{m}$ and for all $\beta \in \pi _{\alpha }$. Indeed, since supp$(\chi _{n})\subseteq M_{2n}^{c}$ we have

5.4 $$\begin{aligned} ||D_{v}^{\beta }f\partial ^{\alpha -\beta }\chi _{n}||_{L^{p}(\Omega )}= &   |||v|^{-s(\beta )-1}\partial ^{\alpha -\beta }\chi _{n}|v|^{s(\beta )+1}D_{v}^{\beta }f||_{L^{p}(\Omega )}\nonumber \\\le &   (2n)^{s(\beta )+1}||\partial ^{\alpha -\beta }\chi _{n}||_{L^{\infty }(\Omega )}|||v|^{s(\beta )+1}D_{v}^{\beta }f||_{L^{p}(\Omega )}.\qquad \end{aligned}$$

We claim now that $\chi _{n}f$ is $\alpha$-weakly differentiable in the sense of $L_{\textrm{loc}}^{1}(\Omega )$ for all $\alpha \in \pi _{m}^{0}$ and that (5.3) holds. Indeed, we will prove this by induction as we did for (4.6). Suppose first that $\alpha \in \pi _{1}^{0}$. Choose $\varphi \in {\mathcal {D}}(\Omega )$. Using (3.2) with the test function $\varphi \chi _{n}v^{-|\alpha |-1}\in {\mathcal {C}}_{c}^{m}(\Omega )$ (because $\chi _{n}\equiv 0$ on $M_{2n}$) we get

$$\begin{aligned} \int _{\Omega }f\partial ^{\alpha }(\chi _{n}\varphi )\,\textrm{d}x=-\int _{\Omega }\chi _{n}\varphi D_{v}^{\alpha }f\,\textrm{d}x. \end{aligned}$$

It follows that

$$\begin{aligned} \int _{\Omega }f\chi _{n}\partial ^{\alpha }\varphi \,\textrm{d}x=\int _{\Omega }f(\partial ^{\alpha }(\chi _{n}\varphi )-\varphi \partial ^{\alpha }\chi _{n})\,\textrm{d}x=-\int _{\Omega }(\chi _{n}D_{v}^{\alpha }f+f\partial ^{\alpha }\chi _{n})\varphi \,\textrm{d}x, \end{aligned}$$

which proves our claim when $\alpha \in \pi _{1}^{0}$. We assume now that (5.3) holds for all $\alpha \in \pi _{l}^{0}$ for some $l<m$. Choose now $\alpha \in \pi _{l+1}^{l}$. Then $\alpha =\beta +\gamma$ for some $|\beta |=l$ and $|\gamma |=1$. Then for $\varphi$ as above,

$$\begin{aligned} \int _{\Omega }f\chi _{n}\partial ^{\alpha }\varphi \,\textrm{d}x= &   (-1)^{|\beta |}\int _{\Omega }D^{\beta }(f\chi _{n})\partial ^{\gamma }\varphi \,\textrm{d}x\\= &   (-1)^{|\beta |}\sum _{0\le \sigma \le \beta }{\beta \atopwithdelims ()\sigma }\int _{\Omega }D_{v}^{\sigma }f\partial ^{\beta -\sigma }\chi _{n}\partial ^{\gamma }\varphi \,\textrm{d}x \end{aligned}$$

where we used the induction assumption. Using the equality

$$\begin{aligned} \partial ^{\beta -\sigma }\chi _{n}\partial ^{\gamma }\varphi =\partial ^{\gamma }(\varphi \partial ^{\beta -\sigma }\chi _{n})-\varphi \partial ^{\alpha -\sigma }\chi _{n} \end{aligned}$$

we get

$$\begin{aligned} \int _{\Omega }f\chi _{n}\partial ^{\alpha }\varphi \,\textrm{d}x= &   (-1)^{|\beta |}\sum _{0\le \sigma \le \beta }{\beta \atopwithdelims ()\sigma }\int _{\Omega }D_{v}^{\sigma }f\partial ^{\gamma }(\varphi \partial ^{\beta -\sigma }\chi _{n})\,\textrm{d}x\\  &   +(-1)^{|\alpha |}\sum _{0\le \sigma \le \beta }{\beta \atopwithdelims ()\sigma }\int _{\Omega }\varphi D_{v}^{\sigma }f\partial ^{\alpha -\sigma }\chi _{n}\,\textrm{d}x. \end{aligned}$$

Again, using (3.2) we obtain

$$\begin{aligned} \int _{\Omega }D_{v}^{\sigma }f\partial ^{\gamma }(\varphi \partial ^{\beta -\sigma }\chi _{n})\,\textrm{d}x=-\int _{\Omega }\varphi \partial ^{\beta -\sigma }\chi _{n}D_{v}^{\gamma +\sigma }f\,\textrm{d}x \end{aligned}$$

Thus

$$\begin{aligned} \int _{\Omega }f\chi _{n}\partial ^{\alpha }\varphi \,\textrm{d}x\!=\!(-1)^{|\alpha |}\!\sum _{0\le \sigma \le \beta }{\beta \atopwithdelims ()\sigma }\int _{\Omega }\varphi (D_{v}^{\rho }f\partial ^{\alpha -\rho }\chi _{n}\!+\!D_{v}^{\sigma }f\partial ^{\alpha -\sigma }\chi _{n})\,\textrm{d}x, \end{aligned}$$

where $\rho =\sigma +\gamma$. It follows that

$$\begin{aligned} \int _{\Omega }f\chi _{n}\partial ^{\alpha }\varphi \,\textrm{d}x=(-1)^{|\alpha |}\sum _{0\le \sigma \le \alpha }{\alpha \atopwithdelims ()\sigma }\int _{\Omega }\varphi (D_{v}^{\sigma }f\partial ^{\alpha -\sigma }\chi _{n})\,\textrm{d}x, \end{aligned}$$

since

$$\begin{aligned} {\beta \atopwithdelims ()\sigma -\gamma }+{\beta \atopwithdelims ()\sigma }={\alpha \atopwithdelims ()\sigma }, \end{aligned}$$

Therefore, the map $W_{s,v}^{m,p}(\Omega )\longrightarrow W^{m,p}(\Omega )$, $f\longrightarrow \chi _{n}f$ is well defined and (5.3) holds. Moreover, the continuity follows from (5.3) and (5.4), completing the proof. $\square$

### Remark 14

Using Theorem 13 with $m=0$ we get that $\chi _{n}f\in L^{p}(\Omega )$. By the dominated convergence theorem we conclude that $\chi _{n}f\longrightarrow f$ in $L_{v}^{p}(\Omega )$ and hence $L^{p}(\Omega )$ is dense in $L_{v}^{p}(\Omega )$. If *v* is bounded on $\Omega$, then ${\mathcal {D}}(\Omega )$ is dense in $L_{v}^{p}(\Omega )$.

Another way to prove the density of ${\mathcal {D}}(\Omega )$ in $L_{w}^{p}(\Omega )$ ($w\in E_{p}(\Omega )$) is to assume that $w\in {\mathcal {C}}^{\infty }(\Omega )$. In this setting, given a sequence $(\varphi _{n})$ of ${\mathcal {D}}(\Omega )$ that converges to *wf* in $L^{2}(\Omega )$, the sequence $(\varphi _{n}\chi _{n}w^{-1})$ ($\chi _{n}$ defined with $v=w$) of ${\mathcal {D}}(\Omega )$ converges to *f* in $L_{w}^{p}(\Omega )$.

The aim now is to show that the sequence $(\chi _{n}f)$ of $W^{m,p}(\Omega )$ (provided assumption (5.2) holds) converges to *f* in $W_{s,v}^{m,p}(\Omega )$ when *v* is bounded. More precisely, we plan to show that

$$\begin{aligned} \forall \alpha \in \pi _{m}:\,|v|^{s(\alpha )+1}D^{\alpha }(f\chi _{n})=|v|^{s(\alpha )+1}\sum _{0\le \beta \le \alpha }{\alpha \atopwithdelims ()\beta }D_{v}^{\beta }f\partial ^{\alpha -\beta }\chi _{n}\rightarrow |v|^{s(\alpha )+1}D_{v}^{\alpha }f \end{aligned}$$

with convergence in $L^{p}(\Omega )$, where we used (5.3) in the first equality. Since $\chi _{n}|v|^{s(\beta )+1}D_{v}^{\beta }f\longrightarrow |v|^{s(\beta )+1}D_{v}^{\beta }f$ in $L^{p}(\Omega )$ for all $\beta \in \pi _{m}$ by the dominated convergence theorem, it suffices to show that

$$\begin{aligned} \forall \alpha \in \pi _{m}\,\forall \beta \in \pi _{\alpha }\backslash \{\alpha \}:\,|v|^{s(\beta )+1}(D_{v}^{\beta }f)\,|v|^{s(\alpha )-s(\beta )}\partial ^{\alpha -\beta }\chi _{n}\longrightarrow 0\,\,\text {in}\,\,L^{p}(\Omega ). \end{aligned}$$

However, since

(i) $\partial ^{\sigma }\chi _{n}\longrightarrow 0$ pointwise a.e. in $\Omega$ for all $\sigma \in \pi _{m}^{0}$,
(ii) $\,|v|^{s(\beta )+1}(D_{v}^{\beta }f)\in L^{p}(\Omega )$ for all $\beta \in \pi _{m}$,
(iii) $\partial ^{\sigma }\chi _{n}=0$ on $M_{n}^{c}\cup M_{2n}$ and $|v|^{s(\sigma )}\le n^{-s(\sigma )}$ on $M_{n}\cap M_{2n}^{c}$ for all $\sigma \in \pi _{m}^{0}$,
(iv) $s(\alpha )-s(\beta )\ge s(\alpha -\beta )$ and hence $|v|^{s(\alpha )-s(\beta )}\le C|v|^{s(\alpha -\beta )}$ for all $x\in \Omega$ for some *C* independent of *x* (since *v* is bounded on $\Omega$)

it suffices to have the following property

5.5 $$\begin{aligned} \exists C>0\,\forall n\in \mathbb {N}\,\forall \sigma \in \pi _{m}^{0}\,\forall x\in M_{n}\cap M_{2n}^{c}:\,|\partial ^{\sigma }\chi _{n}|\le Cn^{s(\sigma )} \end{aligned}$$

and conclude then by the dominated convergence theorem.

In the next lemma we give an assumption on *v* that yields (5.5).

### Lemma 15

Let $\Omega \subseteq \mathbb {R}^{d}$ be an open set, $p\in [1,\infty )$, $m\in \mathbb {N}$, $v\in {\mathcal {C}}^{m,*}(\Omega )$, $s:\pi _{m}\longrightarrow \mathbb {R}_{\ge 0}$ be a map satisfying (4.5). Assume that

5.6 $$\begin{aligned} \exists C>0\,\forall n\in \mathbb {N}\,\forall \sigma \in \pi _{m}^{0}\,\forall x\in M_{n}\cap M_{2n}^{c}:\,|\partial ^{\sigma }v(x)|\le Cn^{s(\sigma )-1}. \end{aligned}$$

Then, (5.5) holds.

### Proof

Fix $n\in \mathbb {N}$ and let $\sigma \in \pi _{m}^{0}$. By the Faà di Bruno formula ( [7]), for any $x\in M_{n}\cap M_{2n}^{c}$ we have

$$\begin{aligned} \partial ^{\sigma }\chi _{n}(x)=\sum _{k=1}^{|\sigma |}|\{\pi \in \Pi \mid |\pi |=k\}|\eta _{n}^{(k)}(v(x))\sum _{\pi \in \Pi ,|\pi |=k}\prod _{B\in \pi }\frac{\partial ^{|B|}v}{\prod _{j\in B}\partial x_{j}}. \end{aligned}$$

(See the proof of (ii) of Proposition 7 for more details). By construction of the sequence $(\eta _{n})$ we have

$$\begin{aligned} \forall k\in \mathbb {N}\,\exists C>0\,\forall n\in \mathbb {N}\,\forall x\in \mathbb {R}:\,|\eta _{n}^{(k)}(x)|\le Cn^{k}. \end{aligned}$$

For any $B\in \pi$, we associate $\beta \in \mathbb {N}^{d}$ by setting $\beta _{j}:=|\{j\in B\}|$ for all *j*. It follows that

5.7 $$\begin{aligned} |\partial ^{\sigma }\chi _{n}(x)|\le C\sum _{k=1}^{|\sigma |}C_{k}n^{k}\sum _{\pi \in \Pi ,|\pi |=k}\prod _{B\in \pi }n^{s(\beta )-1} \end{aligned}$$

where we used assumption (5.6). It is easy to see that

$$\begin{aligned} \forall \pi \in \Pi :\,\prod _{B\in \pi }n^{s(\beta )-1}=n^{\sum _{B\in \pi }s(\beta )-|\pi |}\le n^{s(\sigma )-|\pi |} \end{aligned}$$

where we used (4.5), which gives (5.5) when used in (5.7). $\square$

### Theorem 16

Let $\Omega \subseteq \mathbb {R}^{d}$ be an open set, $p\in [1,\infty )$, $m\in \mathbb {N}$, and $v\in {\mathcal {C}}^{m,*}(\Omega )\cap L^{\infty }(\Omega )$, $s:\pi _{m}\longrightarrow \mathbb {R}_{\ge 0}$ be a map satisfying (4.5). Assume that (5.6) holds. Then, for any $f\in W_{s,v}^{m,p}(\Omega )$, the sequence $(\chi _{n}f)$ of $W^{m,p}(\Omega )$ (by Theorem 13) converges to *f* in $W_{s,v}^{m,p}(\Omega )$.

### Proof

It follows from Lemma 15 and the paragraph preceding it. $\square$

We deduce from this theorem the following density results

### Corollary 17

Under the assumptions of Theorem 16, we have

(i) The space ${\mathcal {D}}(\Omega )$ is dense in $W_{s,v}^{m,p}(\Omega )$ when $\Omega =\mathbb {R}^{d}$.
(ii) The space ${\mathcal {C}}^{\infty }(\Omega )\cap W^{m,p}(\Omega )$ is dense in $W_{s,v}^{m,p}(\Omega )$ in case $\Omega$ is bounded.

### Proof

Let $f\in W_{s,v}^{m,p}(\mathbb {R}^{d})$. By Theorem 16, the sequence $(\chi _{n}f)$ of $W^{m,p}(\mathbb {R}^{d})$ converges to *f* in $W_{s,v}^{m,p}(\mathbb {R}^{d})$. By density of ${\mathcal {D}}(\mathbb {R}^{d})$ in $W^{m,p}(\mathbb {R}^{d})$, for all *n* there exists a sequence $(\varphi _{n,k})_{k}$ of ${\mathcal {D}}(\mathbb {R}^{d})$ that converges to $\chi _{n}f$ in $W^{m,p}(\mathbb {R}^{d})$, and hence in $W_{s,v}^{m,p}(\mathbb {R}^{d})$ since $v\in L^{\infty }(\mathbb {R}^{d})$, which proves assertion (i).

Similarly, to prove assertion (ii), we use Theorem 16 and the fact that the space ${\mathcal {C}}^{\infty }(\Omega )\cap W^{m,p}(\Omega )$ is dense in $W^{m,p}(\Omega )$ when $\Omega$ is bounded (see Thm. 2 of Subsection 5.3.2 of [3]). $\square$

### Remark 18

The boundedness assumption of *v* in Theorem 16 can be dropped in case $s(\alpha )=|\alpha |$ since the assumption was used to guarantee that the constant *C* in (4.1) is independent of *K*.

## The trace operator

Now, we discuss the different possibilities to define a trace operator on $W_{s,v}^{1,p}(\Omega )$

### Remark 19

Let $\Omega \subseteq \mathbb {R}^{d}$ be a bounded open set with a sufficiently smooth boundary,^1^ e.g. $\partial \Omega$ is ${\mathcal {C}}^{1}$, $p\in [1,\infty )$, $v\in {\mathcal {C}}^{1,*}(\Omega )$, $s:\pi _{1}\longrightarrow \mathbb {R}_{\ge 0}$ be a map satisfying (4.5).

(i) Assume that
  6.1 $$\begin{aligned} \exists K\Subset \Omega \,\exists \delta>0\,\forall x\in \Omega \backslash K:\,|v(x)|>\delta \end{aligned}$$
  (or equivalently $M_{n_{0}}\Subset \Omega$ for some $n_{0}\in \mathbb {N}$). Fix $n\ge n_{0}$. Then, (5.2) holds and $\partial \Omega \subseteq \partial M_{n}^{c}$. By Theorem 13 we have
  6.2 $$\begin{aligned} \chi _{n}f\in W^{1,p}(\Omega )\,\,\,\text {and}\,\,\,\forall \alpha \in \pi _{1}^{0}:\,D^{\alpha }(\chi _{n}f)=f\partial ^{\alpha }\chi _{n}+\chi _{n}D_{v}^{\alpha }f. \end{aligned}$$
  If we denote by $|_{\partial \Omega }:W^{1,p}(\Omega )\longrightarrow L^{p}(\partial \Omega )$ the trace operator, then $(f\chi _{n})|_{\partial \Omega }$ is well defined. Since $\chi _{n}\equiv 1$ on $\Omega \backslash K\subseteq M_{n}^{c}$, we have that $(f\chi _{n})|_{\partial \Omega }=f|_{\partial \Omega }$ which implies that $|_{\partial \Omega }$ can be extended to the space $W_{s,v}^{1,p}(\Omega )$. Furthermore, by Theorem 13 and the trace theorem in $W^{1,p}(\Omega )$ we get
  6.3 $$\begin{aligned} ||f|_{\partial \Omega }||_{L^{p}(\partial \Omega )}\le C||\chi _{n}f||_{W^{1,p}(\Omega )}\le C_{n}||f||_{W_{s,v}^{1,p}(\Omega )} \end{aligned}$$
  where $C_{n}$ is a constant that depends on *n* but not in *f*, which proves that $|_{\partial \Omega }$ is continuous on $W_{s,v}^{1,p}(\Omega )$.
(ii) If we assume that $v\in {\mathcal {C}}_{b}^{1}(\Omega )$, then by Proposition 12, we have that the map $W_{s,v}^{1,p}(\Omega )\longrightarrow W^{1,p}(\Omega )$, $f\longrightarrow v^{2}f$ is continuous. In particular $(v^{2}f)|_{\partial \Omega }$ is well defined and belongs to $L^{p}(\partial \Omega )$. Moreover, by the trace theorem in $W^{1,p}(\Omega )$ we have
  6.4 $$\begin{aligned} ||(v^{2}f)|_{\partial \Omega }||_{L^{p}(\partial \Omega )}\le C||v^{2}f||_{W^{1,p}(\Omega )}\le C||f||_{W_{s,v}^{1,p}(\Omega )} \end{aligned}$$
  Therefore, the operator $W_{s,v}^{1,p}(\Omega )\longrightarrow L^{p}(\partial \Omega )$, $f\longrightarrow (v^{2}f)|_{\partial \Omega }$ is continuous.

Therefore, we can define the trace operator on the space $W_{s,v}^{1,p}(\Omega )$ either by setting $\text {Tr}(f)=f|_{\partial \Omega }\in L^{p}(\partial \Omega )$, or by setting $\text {Tr}(f)=(v^{2}f)|_{\partial \Omega }\in L^{p}(\partial \Omega )$ depending on the assumptions satisfied by the function *v*.

### Remark 20

Note that in the favorite situation in which assumption (6.1) holds and $v\in {\mathcal {C}}_{b}^{1}(\Omega )$, both $f|_{\partial \Omega }$ and $(v^{2}f)|_{\partial \Omega }$ are well defined and belong to $L^{p}(\partial \Omega )$ for all $f\in W_{s,v}^{1,p}(\Omega )$. Moreover, both *v* and $v^{-1}$ are bounded on $\partial \Omega$, which implies that the operators $W_{s,v}^{1,p}(\Omega )\longrightarrow L^{p}(\partial \Omega )$, $f\longrightarrow f|_{\partial \Omega }$, $f\longrightarrow (fv^{2})|_{\partial \Omega }$ are equivalent, and hence $f|_{\partial \Omega }=0$ if and only if $(v^{2}f)|_{\partial \Omega }=0$.

These different possibilities motivate the following definition

### Definition 21

Let $\Omega \subseteq \mathbb {R}^{d}$ be a bounded open set and $\partial \Omega$ is ${\mathcal {C}}^{1}$, $p\in [1,\infty )$, $v\in {\mathcal {C}}^{1,*}(\Omega )$, $s:\pi _{1}\longrightarrow \mathbb {R}_{\ge 0}$ be a map satisfying (4.5).

(i) If assumption (6.1) holds, then
  6.5 $$\begin{aligned} \forall f\in W_{s,v}^{1,p}(\Omega ):\,\text {Tr}_{1}(f):=f|_{\partial \Omega }\in L^{p}(\partial \Omega ) \end{aligned}$$
(ii) If $v\in {\mathcal {C}}_{b}^{1}(\Omega )$, then
  6.6 $$\begin{aligned} \forall f\in W_{s,v}^{1,p}(\Omega ):\,\text {Tr}_{2}(f):=(v^{2}f)|_{\partial \Omega }\in L^{p}(\partial \Omega ). \end{aligned}$$

As we have seen in the beginning of this section, under the assumptions given in the latter definition, the trace operators are continuous (see (6.3) and (6.4)).

## Spaces of null trace

### Definition 22

Let $\Omega \subseteq \mathbb {R}^{d}$ be an open set, $p\in [1,\infty )$, $V:=\{w_{\alpha }\}_{\alpha \in \pi _{1}}$ be a collection of elements of $E_{p}(\Omega )$, and let $v\in {\mathcal {C}}^{1,*}(\Omega )$ be such that (4.1) holds; $X_{V,v,0}^{1,p}(\Omega )$ denotes the closure of ${\mathcal {D}}(\Omega )$ in $W_{V,v}^{1,p}(\Omega )$.

The assumption (4.1) guarantees that the weighted Sobolev space $W_{V,v}^{1,p}(\Omega )$ is Cauchy complete, and hence $X_{V,v,0}^{1,p}(\Omega )$ is well defined.

We now define the spaces $W_{s,v,0}^{1,p,i}$ ($i=1,$ 2) using the trace operators

### Definition 23

Let $\Omega$ be a bounded open set and $\partial \Omega$ is ${\mathcal {C}}^{1}$, $p\in [1,\infty )$, $v\in {\mathcal {C}}^{1,*}(\Omega )$, $s:\pi _{1}\longrightarrow \mathbb {R}_{\ge 0}$ be a map satisfying (4.5).

(i) Assume that (6.1) holds. Then we set
  $$\begin{aligned} W_{s,v,0}^{1,p,1}(\Omega ):=\{f\in W_{s,v}^{1,p}(\Omega )\mid \text {Tr}_{1}(f)=0\}. \end{aligned}$$
(ii) Assume that $v\in {\mathcal {C}}_{b}^{1}(\Omega )$. Then we set
  $$\begin{aligned} W_{s,v,0}^{1,p,2}(\Omega ):=\{f\in W_{s,v}^{1,p}(\Omega )\mid \text {Tr}_{2}(f)=0\}. \end{aligned}$$

### Remark 24

 Recall from Remark 20 that the operators $\text {Tr}_{1}$, $\text {Tr}_{2}$ are equivalent when $v\in {\mathcal {C}}_{b}^{1}(\Omega )$ and (6.1) holds, which implies that

$$\begin{aligned} W_{s,v,0}^{1,p,1}(\Omega )=W_{s,v,0}^{1,p,2}(\Omega ). \end{aligned}$$

### Proposition 25

Let $\Omega$ be a bounded open set and $\partial \Omega$ is ${\mathcal {C}}^{1}$, $p\in [1,\infty )$, $v\in {\mathcal {C}}^{1,*}(\Omega )$, $s:\pi _{1}\longrightarrow \mathbb {R}_{\ge 0}$ be a map satisfying (4.5).

(i) If (6.1) holds, then the space $W_{s,v,0}^{1,p,1}(\Omega )$ is Cauchy complete.
(ii) If $v\in {\mathcal {C}}_{b}^{1}(\Omega )$, then the space $W_{s,v,0}^{1,p,2}(\Omega )$ is Cauchy complete.

### Proof

It is sufficient to prove that the space $W_{s,v,0}^{1,p,i}(\Omega )$ ($i=1$, 2) is closed since it is a subspace of a Banach space $W_{s,v}^{1,p}(\Omega )$. However, the closedness follows from the fact that $W_{s,v,0}^{1,p,i}(\Omega )=\textrm{Ker}(\textrm{Tr}_{i})$ and $\text {Tr}_{i}$ is continuous. $\square$

### Theorem 26

Let $\Omega$ be a bounded open set and $\partial \Omega$ is ${\mathcal {C}}^{1}$, $p\in [1,\infty )$, $v\in {\mathcal {C}}^{1,*}(\Omega )$, $s:\pi _{1}\longrightarrow \mathbb {R}_{\ge 0}$ be a map satisfying (4.5).

(i) If *v* is bounded and (6.1) holds, then $X_{s,v,0}^{1,p}(\Omega )=W_{s,v,0}^{1,p,1}(\Omega ).$
(ii) If $v\in {\mathcal {C}}_{b}^{1}(\Omega )$ then $X_{s,v,0}^{1,p}(\Omega )=W_{s,v,0}^{1,p,2}(\Omega ).$

### Proof

For $i=1$, 2, the inclusion $X_{s,v,0}^{1,p}(\Omega )\subseteq W_{s,v,0}^{1,p,i}(\Omega )$ follows from the fact that $\text {Tr}_{i}(\varphi )=0$ for all $\varphi \in {\mathcal {D}}(\Omega )$, and $\text {Tr}_{i}$ is continuous.

We deal now with the opposite inclusion. Claim first that $\chi _{n}f\in W_{0}^{1,p}(\Omega )$ and that the sequence $(\chi _{n}f)$ converges to *f* in $W_{s,v}^{1,p}(\Omega )$ for all $f\in W_{s,v,0}^{1,p,i}(\Omega )$ ($i=1$, 2). Indeed, in case (6.1) holds, the first part of the claim follows from the definition of the space $W_{s,v,0}^{1,p,1}(\Omega )$ (see also (i) of Remark 19). In case $v\in {\mathcal {C}}_{b}^{1}(\Omega )$, $\chi _{n}f\in W^{1,p}(\Omega )$ by Theorem (13). Moreover, we have that $(f\chi _{n})|_{\partial M_{2n}\cap \partial \Omega }=0$ because $\chi _{n}\equiv 0$ on $M_{2n}$, and $(f\chi _{n})|_{\partial M_{2n}^{c}\cap \partial \Omega }=(fv^{2}\chi _{n}v^{-2})|_{\partial M_{2n}^{c}\cap \partial \Omega }=0$ because $|v^{-2}|\le (2n)^{2}$ on $M_{2n}^{c}$, which proves that $f\chi _{n}\in W_{0}^{1,p}(\Omega )$, and hence the first part of the claim is proved. The second part of the claim is a consequence of Theorem 16. Finally, by density of ${\mathcal {D}}(\Omega )$ in $W_{0}^{1,p}(\Omega )$, and since *v* is bounded, we conclude that ${\mathcal {D}}(\Omega )$ is dense $W_{s,v,0}^{1,p,i}(\Omega )$. $\square$

In the next lemma, we consider a functional of the form $X_{s,v,0}^{1,p}(\Omega )\longrightarrow \mathbb {R}$, $f\longrightarrow \int _{\Omega }gD^{\alpha }(v^{3}f)\,\textrm{d}x$ where $v\in {\mathcal {C}}^{1,*}(\Omega )$, $s:\pi _{1}\longrightarrow \mathbb {R}_{\ge 0}$ is a map satisfying (4.5), and $g\in W_{s,v}^{1,p'}(\Omega )$ ($p'$ is the exponent conjugate to *p*). We show in particular that

7.1 $$\begin{aligned} \forall f\in X_{s,v,0}^{1,p}(\Omega )\,\forall \alpha \in \pi _{1}^{0}:\,\int _{\Omega }gD^{\alpha }(v^{3}f)\,\textrm{d}x=-\int _{\Omega }v^{3}fD_{v}^{\alpha }g\,\textrm{d}x \end{aligned}$$

which implies that the functional $f\longrightarrow \int _{\Omega }gD^{\alpha }(v^{3}f)\,\textrm{d}x$ is continuous. As far as we know, the latter functional and the integration by part formula (7.1) was not considered in the literature.

### Lemma 27

Let $\Omega \subseteq \mathbb {R}^{d}$ be a bounded open set, $v\in {\mathcal {C}}^{1,*}(\Omega )$, $s:\pi _{1}\longrightarrow \mathbb {R}_{\ge 0}$ be a map satisfying (4.5). Assume that

(i) $$v\in L^{\infty }(\Omega )$$
(ii) $a\in L_{v^{-3}}^{\infty }(\Omega )$ with $av^{-2}\in {\mathcal {C}}^{1}(\Omega )$ and $\partial ^{\alpha }a\in L_{v^{-2}}^{\infty }(\Omega )$ for all $\alpha \in \pi _{1}^{0}$.

Then

$$\begin{aligned} \forall q\in [1,\infty )\,\forall g\in W_{s,v}^{1,q}(\Omega ):\,ag\in W^{1,q}(\Omega )\,\,\,\text {and}\,\,\,D^{\alpha }(ag)=g\partial ^{\alpha }a+aD_{v}^{\alpha }g. \end{aligned}$$

Moreover,

7.2 $$\begin{aligned} \forall h\in W_{s,v}^{1,p}(\Omega )\,\forall f\in X_{s,v,0}^{1,p'}(\Omega ):\int _{\Omega }hD^{\alpha }(af)\,\textrm{d}x=-\int _{\Omega }afD_{v}^{\alpha }h\,\textrm{d}x \end{aligned}$$

for all $p\in (1,\infty )\,$, where $p'$ is the exponent conjugate to *p*.

### Proof

The proof of the first part of the lemma is very similar to the proof of Theorem 12. We prove now (7.2). By definition of $X_{s,v,0}^{1,p'}$, there exists a sequence $(\varphi _{n})$ of ${\mathcal {D}}(\Omega )$ that converges to *f* in $W_{s,v}^{1,p'}(\Omega )$. Since $h\in W_{s,v}^{1,p}(\Omega )$, we have

$$\begin{aligned} \int _{\Omega }h\varphi _{n}\partial ^{\alpha }a\,\textrm{d}x+\int _{\Omega }ha\partial ^{\alpha }\varphi _{n}\,\textrm{d}x=\int _{\Omega }h\partial ^{\alpha }(a\varphi _{n})\,\textrm{d}x=-\int _{\Omega }a\varphi _{n}D_{v}^{\alpha }h\,\textrm{d}x \end{aligned}$$

for all *n* and for all $\alpha \in \pi _{1}^{0}$, where we simply used the definition of the weak derivative in the sense of $L_{v,\textrm{loc}}^{1}(\Omega )$ with the test function $av^{-2}\varphi _{n}\in {\mathcal {C}}_{0}^{1}(\Omega )$. By letting *n* tends to $\infty$ we get

7.3 $$\begin{aligned} \int _{\Omega }hf\partial ^{\alpha }a\,\textrm{d}x+\int _{\Omega }haD_{v}^{\alpha }f\,\textrm{d}x=-\int _{\Omega }afD_{v}^{\alpha }h\,\textrm{d}x \end{aligned}$$

since

$$\begin{aligned}  &   \left| \int _{\Omega }h\partial ^{\alpha }a(\varphi _{n}-f)\,\textrm{d}x\right| \le C||\partial ^{\alpha }a||_{L_{v^{-2}}^{\infty }(\Omega )}||hv||_{L^{p}(\Omega )}||v(\varphi _{n}-f)||_{L^{p'}(\Omega )},\\  &   \left| \int _{\Omega }ha(\partial ^{\alpha }\varphi _{n}-D_{v}^{\alpha }f)\,\textrm{d}x\right| \!\le \! C||a||_{L_{v^{-3}}^{\infty }(\Omega )}||hv||_{L^{p}(\Omega )}||(\partial ^{\alpha }\varphi _{n}\!-\!D_{v}^{\alpha }f)|v|^{s(\alpha )+1}||_{L^{p'}(\Omega )},\quad \end{aligned}$$

and

$$\begin{aligned} \left| \int _{\Omega }a(\varphi _{n}-f)D_{v}^{\alpha }h\,\textrm{d}x\right| \le C||a||_{L_{v^{-3}}^{\infty }(\Omega )}|||v|^{s(\alpha )+1}D_{v}^{\alpha }h||_{L^{p}(\Omega )}||v(\varphi _{n}-f)||_{L^{p'}(\Omega )}. \end{aligned}$$

Using now (7.3) and the first part of the lemma with $g=f$ we obtain (7.2). $\square$

### Remark 28

(i) When $v\in {\mathcal {C}}_{b}^{1}(\Omega )$, the function *a* in the previous lemma can be any function of the form $a={\tilde{a}}v^{n}$ where ${\tilde{a}}\in {\mathcal {C}}_{b}^{1}(\Omega )$ and $n\in \mathbb {N}$ with $n\ge 3$.
(ii) Clearly, the first part of the lemma can be proved with less assumptions on $\Omega$, *v* and *a*.

## Poincaré inequality

In this section we are interesting in proving a Poincaré inequality for the weighted Sobolev space $X_{s,v,0}^{1,p}(\Omega )$.

We first derive an interesting inequality where we prove that the term $||f\nabla (v^{2})||_{L^{p}}$ is bounded by $||v^{2}D_{v}f||_{L^{p}}$. We use then this inequality to prove an interesting Poincaré inequality under the assumption $v\in {\mathcal {C}}_{b}^{1,*}(\Omega ):={\mathcal {C}}_{b}^{1}(\Omega )\cap {\mathcal {C}}^{1,*}(\Omega )$.

### Theorem 29

Let $\Omega \subseteq \mathbb {R}^{d}$ ($d\ge 2$) be a bounded open set, $p\in [1,d)$, $v\in {\mathcal {C}}_{b}^{1,*}(\Omega )$, $s:\pi _{1}\longrightarrow \mathbb {R}_{\ge 0}$ be a map satisfying (4.5). Assume that

8.1 $$\begin{aligned} \exists \sigma >0\,\forall x\ne 0:\,|\nabla v(x)|_{p}:=\left( \sum _{i=1}^{d}|\partial _{i}v(x)|^{p}\right) ^{1/p}\le \sigma \frac{|v(x)|}{|x|} \end{aligned}$$

where |*x*| is the euclidean norm of $\mathbb {R}^{d}$, and that

8.2 $$\begin{aligned} 0<\frac{2\sigma p}{d-p}<1. \end{aligned}$$

Then,

8.3 $$\begin{aligned} \forall f\in X_{s,v,0}^{1,p}(\Omega ):\,||f\nabla (v^{2})||_{L^{p}}\le \frac{2\sigma p}{d-p-2\sigma p}||v^{2}D_{v}f||_{L^{p}}. \end{aligned}$$

### Proof

Let $f\in X_{s,v,0}^{1,p}(\Omega )$. By Theorem 26 we have

$$\begin{aligned} fv^{2}\in W_{0}^{1,p}(\Omega )\,\text {with }D^{\alpha }(fv^{2})=v^{2}D_{v}^{\alpha }f+f\partial ^{\alpha }v^{2},\,\,\,\forall \alpha \in \pi _{1}^{0}. \end{aligned}$$

(See also Proposition 12.) Using (8.1) and Hardy inequality we get

$$\begin{aligned} ||f\nabla (v^{2})||_{L^{p}(\Omega )}&:=2\left( \sum _{i=1}^{d}||fv\partial _{i}v||_{L^{p}(\Omega )}^{p}\right) ^{\frac{1}{p}}\\&\le 2\sigma \left\| \frac{fv^{2}}{x}\right\| _{L^{p}(\Omega )}\\&\le 2\sigma \frac{p}{d-p}\left\| D(fv^{2})\right\| _{L^{p}(\Omega )}\\&\le 2\sigma \frac{p}{d-p}\left\| v^{2}D_{v}f\right\| _{L^{p}(\Omega )}+2\sigma \frac{p}{d-p}\left\| f\nabla (v^{2})\right\| _{L^{p}(\Omega )} \end{aligned}$$

This inequality together with (8.2) gives (8.3). $\square$

### Example 30

The typical example of a function satisfying (8.1) when $p\le 2$ is the function $v(x)=|x|^{\beta }$ with $\beta \ge 2$, where $\sigma =\beta d^{\frac{2-p}{2}}$.

Similarly, we have

### Theorem 31

Let $\Omega \subseteq \mathbb {R}$ be an open set, $p\in (1,\infty )$, $v\in {\mathcal {C}}^{1,*}(\Omega )$ with $v'\in L^{\infty }(\Omega )$, and let $s:\pi _{1}\longrightarrow \mathbb {R}_{\ge 0}$ be a map satisfying (4.5). Assume that

$$\begin{aligned} \exists \sigma >0\,\forall x\ne 0:\,|v'(x)|\le \sigma \frac{|v(x)|}{|x|},\,\,\,0<\frac{2\sigma p}{p-1}<1. \end{aligned}$$

Then

$$\begin{aligned} \forall f\in X_{s,v,0}^{1,p}(\Omega ):\,||f(v^{2})'||_{L^{p}(\Omega )}\le \frac{2\sigma p}{p-1-2\sigma p}||v^{2}D_{v}f||_{L^{p}(\Omega )}. \end{aligned}$$

The proof is identical to the proof of Theorem 29.

Using Poincaré inequality and Theorem 29 we get

### Corollary 32

Let $\Omega \subseteq \mathbb {R}^{d}$ ($d\ge 2$) be a bounded open set, $p\in [1,d)$, $v\in {\mathcal {C}}_{b}^{1,*}(\Omega )$, $s:\pi _{1}\longrightarrow \mathbb {R}_{\ge 0}$ be a map satisfying (4.5). Assume that (8.1) and (8.2) hold. Then,

$$\begin{aligned} \exists C_{\Omega }>0\,\forall f\in X_{s,v,0}^{1,p}:\,||v^{2}f||_{L^{p}}\le C_{\Omega }||v^{2}D_{v}f||_{L^{p}} \end{aligned}$$

### Proof

This is a direct consequence of Poincaré inequality in $W_{0}^{1,p}(\Omega )$ and Theorem 29. $\square$

## Degenerate elliptic PDE

In the sequel, if $\alpha \in \pi _{1}^{0}$, we write $D_{v}^{i}$ instead of $D_{v}^{\alpha }$ where *i* is the index of the non-null component of $\alpha$. We also write $D_{v}$ for the operator $(D_{v}^{i})$.

We consider in this section, a boundary-value problem of the form

9.1 $$\begin{aligned} {\left\{ \begin{array}{ll} -\sum _{i,j=1}^{d}D_{j}(a_{ij}D_{i}f)+(b\cdot D)f+cf=h &  \text {in }\Omega \\ f=0 &  \text {on }\partial \Omega \end{array}\right. } \end{aligned}$$

where the coefficients $(a_{ij})$, $b=(b_{i})$, *c* are given functions, and *h* is some given functional. In particular, we consider that the bilinear form $\sum _{i,j=1}^{d}a_{ij}(x)\xi _{i}\xi _{j}$ degenerates i.e. is not uniformly elliptic. Let $V:=\{w_{\alpha }\}_{\alpha \in \pi _{1}}$ be a collection of elements of $E_{p}(\Omega )$, and $v\in {\mathcal {C}}^{1,*}(\Omega )$ that satisfy (4.1), and *h* be a continuous functional on $X_{V,v,0}^{1}(\Omega )$ ($:=X_{V,v,0}^{1,2}(\Omega )$). We say that $f\in X_{V,v,0}^{1}(\Omega )$ is a weak solution to the boundary-value problem (9.1) if

9.2 $$\begin{aligned} \forall g\in X_{V,v,0}^{1}(\Omega ):\,B_{v}(f,g)=h(g) \end{aligned}$$

where $B_{v}(\cdot ,\cdot ):X_{V,v,0}^{1}(\Omega )\times X_{V,v,0}^{1}(\Omega )\longrightarrow \mathbb {R}$ is the bilinear form given by

9.3 $$\begin{aligned} B_{v}(f,g):=\sum _{i,j=1}^{d}\int _{\Omega }a_{ij}D_{v}^{i}fD_{v}^{j}g\,\textrm{d}x+\int _{\Omega }g(b\cdot D_{v})f\,\textrm{d}x+\int _{\Omega }cfg\,\textrm{d}x. \end{aligned}$$

Assume that $f\in X_{V,v,0}^{1}(\Omega )$ is a weak solution to (9.1) and assume that

9.4 $$\begin{aligned} \forall i,j=1,...,d:\,a_{ij},\,b_{i},\,c\in L_{\textrm{loc}}^{\infty }(\Omega _{|v|^{2p}}^{*})\,\text { and }\,h\in L_{\textrm{loc}}^{2}(\Omega _{|v|^{2p}}^{*}). \end{aligned}$$

Then the differential equation of (9.1) holds in the sense of ${\mathcal {D}}'(\Omega _{|v|^{2p}}^{*})$. Indeed, we know that $X_{V,v,0}^{1}(\Omega )\subseteq W^{1,2}(\Omega _{|v|^{2p}}^{*},V)$. Hence,

$$\begin{aligned} \forall i,j=1,...,d:\,a_{ij}D_{i}f,\,b_{i}D_{i}f,\,cf,\,h\in L_{\text {loc}}^{1}(\Omega _{|v|^{2p}}^{*})\subseteq {\mathcal {D}}'(\Omega _{|v|^{2p}}^{*}). \end{aligned}$$

The remaining part of the proof is trivial.

For an in-depth study of weak solution existence to boundary value problems of the form (9.1), we refer to [6] and to reference therein. In the next theorem, we give sufficient assumptions that guarantee the existence of a unique weak solution in $X_{s,v,0}^{1}(\Omega )$ to the boundary-value problem (9.1). We use in particular the Poincaré inequality proved in Corollary 32. Moreover, it is possible to take $h(g)=\int _{\Omega }k\nabla (v^{3}g)\,\textrm{d}x$ for all $g\in X_{s,v,0}^{1}(\Omega )$ where $k\in W_{s,v}^{1,2}(\Omega )$ (see Lemma 27).

For simplicity, we will limit ourselves to the case where $s(\cdot )=|\cdot |$ (in particular $w_{\alpha }=v^{|\alpha |+1}\in {\mathcal {C}}^{1}(\Omega )$ for all $\alpha$). We will then write $X_{|\cdot |,v,0}^{1}(\Omega )$ instead of $X_{s,v,0}^{1}(\Omega )$. A generalization to an arbitrary map *s* satisfying (4.5), is possible with some small modification of the assumptions.

### Theorem 33

Let $\Omega \subseteq \mathbb {R}^{d}$ be a bounded open set, $v\in {\mathcal {C}}^{1,*}(\Omega )$, $(a_{ij})$ be such that $a_{ij}\in L_{v^{-4}}^{\infty }(\Omega )$ and satisfies

9.5 $$\begin{aligned} \exists \mu >0\,\forall \xi \in \mathbb {R}^{d}:\,\mu v^{4}(x)|\xi |^{2}\le \sum _{i,j=1}^{d}a_{ij}(x)\xi _{i}\xi _{j}\,\,\,\mathrm {a.e.}\,\text {in }\,\Omega . \end{aligned}$$

Let *c* be such that $v^{-2}c\in L^{\infty }(\Omega )$, and *h* a continuous functional on $X_{|\cdot |,v,0}^{1}(\Omega )$. Suppose furthermore that one of the following assumptions holds

(i) $v^{-3}b_{i}\in L^{\infty }(\Omega )$ for all $i=1,...,d$, $cv^{-2}\ge \sigma >0$ a.e. in $\Omega$, and $d^{\frac{1}{2}}||v^{-3}b||_{L^{\infty }}<2\sqrt{\mu \sigma }$, where $||v^{-3}b||_{L^{\infty }}:=\max _{i}||v^{-3}b_{i}||_{L^{\infty }}$.
(ii) $v\in {\mathcal {C}}_{b}^{1}(\Omega )$ satisfies (8.1), (8.2) with $p=2$ (and hence we should have $d\ge 3$), $v^{-4}b_{i}\in L^{\infty }(\Omega )$ for all $i=1,...,d$, $cv^{-2}\ge \sigma >0$ a.e. in $\Omega$, and
  9.6 $$\begin{aligned} 0<\mu -C_{\Omega }d^{\frac{1}{2}}||v^{-4}b||_{L^{\infty }} \end{aligned}$$
(iii) $v^{-3}b_{i}\in L^{\infty }(\Omega )\cap {\mathcal {C}}^{1}(\Omega )$ for all $i=1,...,d$, and one of the following assertions holds
     (a) $\text {div}(b)\le 0$ a.e. in $\Omega$ and $cv^{-2}\ge \sigma >0$ a.e. in $\Omega$
     (b) $$v^{-2}\text {div}(b)\in L^{\infty }(\Omega )$$ and $$(c-\frac{1}{2}\text {div}(b))v^{-2}\ge \sigma >0$$ a.e. in $$\Omega $$.

Then, the boundary-value problem (9.1) has a unique weak solution $f\in X_{|\cdot |,v,0}^{1}(\Omega )$. In addition, we have the estimate

9.7 $$\begin{aligned} \exists \gamma >0:\,||f||_{X_{|\cdot |,v}^{1}}\le \frac{1}{\gamma }|h| \end{aligned}$$

where |*h*| is the norm operator of *h*.

### Proof

We check that the bilinear form $B_{v}$ defined in (9.3) satisfies the assumptions of the Lax-Milgram theorem in the Hilbert space $X_{|\cdot |,v,0}^{1}(\Omega )$. Note that in any cases, we have $b_{i}v^{-3}\in L^{\infty }(\Omega )$. Hence, for any *f*, $g\in X_{|\cdot |,v,0}^{1}(\Omega )$ we have

$$\begin{aligned} \left| B_{v}(f,g)\right|\le &   C\sum _{i,j=1}^{d}||v^{2}D_{v}^{i}f||_{L^{2}}||v^{2}D_{v}^{j}g||_{L^{2}}+C\sum _{i=1}^{d}||v^{2}D_{v}^{i}f||_{L^{2}}||vg||_{L^{2}}\\  &   +C||vf||_{L^{2}}||vg||_{L^{2}}\le C||f||_{X_{|\cdot |,v}^{1}}||g||_{X_{|\cdot |,v}^{1}} \end{aligned}$$

for some non-negative constant *C*, which proves the continuity of the bilinear form $B_{v}$. We prove now that the bilinear form $B_{v}$ is coercive, that is,

9.8 $$\begin{aligned} \exists \gamma >0\,\forall f\in X_{|\cdot |,v,0}^{1}(\Omega ):\,\gamma ||f||_{X_{|\cdot |,v}^{1}}^{2}\le B_{v}(f,f). \end{aligned}$$

Fix $f\in X_{|\cdot |,v,0}^{1}$. Under the assumptions on $(a_{ij})$ we have

$$\begin{aligned} \mu ||v^{2}D_{v}f||_{L^{2}}^{2}+\int _{\Omega }cf^{2}\,\textrm{d}x+\int _{\Omega }f(b\cdot D_{v})f\,\textrm{d}x\le B_{v}(f,f). \end{aligned}$$

Suppose that assertion (i) holds. Then by Hölder’s inequality, we have

9.9 $$\begin{aligned} \mu ||v^{2}D_{v}f||_{L^{2}}^{2}+\sigma ||vf||_{L^{2}}^{2}-d^{\frac{1}{2}}||v^{-3}b||_{L^{\infty }}||v^{2}D_{v}f||_{L^{2}}||vf||_{L^{2}}\le B_{v}(f,f)\nonumber \\ \end{aligned}$$

Thanks to the assumptions of the assertion (i), it is possible to choose $\gamma$ sufficiently small so that $0<\gamma \le \min (\mu ,\sigma )$ and $d^{\frac{1}{2}}||b||_{L_{v^{-3}}^{\infty }}<2\sqrt{(\mu -\gamma )(\sigma -\gamma )}$. Thus, it follows that left hand side of (9.9) is bounded from bellow by $\gamma (||v^{2}D_{v}f||_{L^{2}}^{2}+||vf||_{L^{2}}^{2})$ which gives (9.8).

In case assertion (ii) holds, we use Hölder’s inequality to obtain

$$\begin{aligned} \mu ||v^{2}D_{v}f||_{L^{2}}^{2}+\sigma ||vf||_{L^{2}}^{2}-d^{\frac{1}{2}}||v^{-4}b||_{L^{\infty }}||v^{2}D_{v}f||_{L^{2}}||v^{2}f||_{L^{2}}\le B_{v}(f,f) \end{aligned}$$

By Corollary 32, we have

$$\begin{aligned} (\mu -C_{\Omega }d^{\frac{1}{2}}||v^{-4}b||_{L^{\infty }})||v^{2}D_{v}f||_{L^{2}}^{2}+\sigma ||vf||_{L^{2}}^{2}\le B_{v}(f,f). \end{aligned}$$

Therefore, (9.8) holds with the constant $\gamma :=\min ((\mu -C_{\Omega }d^{\frac{1}{2}}||v^{-4}b||_{L^{\infty }}),\sigma )$ which is non-negative thanks to (9.6).

Assume now that (iii) holds. By (9.5) we have

9.10 $$\begin{aligned} \mu ||v^{2}D_{v}f||_{L^{2}}^{2}+\int _{\Omega }f(b\cdot D_{v})f\,\textrm{d}x+\int _{\Omega }cf^{2}\,\textrm{d}x\le B_{v}(f,f). \end{aligned}$$

Let $(\varphi _{n})$ be a sequence of ${\mathcal {D}}(\Omega )$ that converges to *f* in $X_{|\cdot |,v}^{1}(\Omega )$. Since $v^{-3}b_{i}\in L^{\infty }(\Omega )$ we have

$$\begin{aligned} \left| \int _{\Omega }b_{i}(fD_{v}^{i}f-\varphi _{n}\partial _{i}\varphi _{n})\,\textrm{d}x\right|\le &   ||b_{i}v^{-3}||_{L^{\infty }}||fv||_{L^{2}}||v^{2}(D_{v}^{i}f-\partial _{i}\varphi _{n})||_{L^{2}}\\  &   +||b_{i}v^{-3}||_{L^{\infty }}||v(f-\varphi _{n})||_{L^{2}}||v^{2}\partial _{i}\varphi _{n}||_{L^{2}}. \end{aligned}$$

Thus,

9.11 $$\begin{aligned} \int _{\Omega }f(b\cdot D_{v})f\,\textrm{d}x=\lim _{n\rightarrow \infty }\int _{\Omega }\varphi _{n}(b\cdot \nabla )\varphi _{n}\,\textrm{d}x=-\frac{1}{2}\lim _{n\rightarrow \infty }\int _{\Omega }\text {div}(b)\varphi _{n}^{2}\,\textrm{d}x.\qquad \end{aligned}$$

Hence, in case (a) of (iii) holds, the right hand side of the latter equality is positive. Thus, the left hand side of (9.10) is bounded from bellow by $\gamma ||f||_{X_{|\cdot |,v}^{1}}^{2}$ where $\gamma =\min (\mu ,\sigma )>0$, and hence (9.8) is proved, and in case (b) of (iii) holds, the right hand side of (9.11) is equal to $-\frac{1}{2}\int _{\Omega }\text {div}(b)f^{2}\,\textrm{d}x$. Replacing this in (9.10) we get

$$\begin{aligned} \gamma ||f||_{X_{|\cdot |,v}^{1}}^{2}\le \mu ||v^{2}D_{v}f||_{L^{2}}^{2}+\sigma ||vf||_{L^{2}}^{2}\le B_{v}(f,f) \end{aligned}$$

where $\gamma :=\min (\mu ,\sigma )>0$. Thus, (9.8) holds.

Therefore, if one of the assertions (i)-(iii) holds, the bilinear form $B_{v}$ is coercive. On the other hand, *h* is a continuous functional on $X_{|\cdot |,v,0}^{1}$. We conclude then by the Lax-Milgram theorem.

For the estimate (9.7), it follows easily from (9.2), (9.8), and the continuity of *h*. Indeed, if we denote by *f* the unique weak solution of (9.1), then

$$\begin{aligned} \gamma ||f||_{X_{|\cdot |,v}^{1}}^{2}\le B_{v}(f,f)=h(f)\le |h|||f||_{X_{|\cdot |,v}^{1}}. \end{aligned}$$

$$\square $$

### Example 34

Let $v\in {\mathcal {C}}^{1,*}(\Omega )$, and let $({\tilde{a}}_{ij})$, $({\tilde{b}})=({\tilde{b}}_{i})$, ${\tilde{c}}$ be such that

(i) $$\forall i,j=1,...,d:\,{\tilde{a}}_{ij},\,{\tilde{b}}_{i},\,{\tilde{c}}\in L^{\infty }(\Omega )$$
(ii) $\exists \mu >0$ such that $\sum _{i,j}{\tilde{a}}_{ij}\xi _{i}\xi _{j}\ge \mu |\xi |^{2}$ a.e. in $\Omega$ and for all $\xi \in \mathbb {R}^{d}$
(iii) $\exists \sigma >0$ such that ${\tilde{c}}\ge \sigma$ a.e. in $\Omega$, and $d^{\frac{1}{2}}||{\tilde{b}}||_{L^{\infty }(\Omega )}<2\sqrt{\mu \sigma }$.

Set

$$\begin{aligned} \forall i,j=1,...,d:\,a_{ij}=v^{4}{\tilde{a}}_{ij},\,b_{i}=v^{\text {3}}{\tilde{b}}_{i},\,c=v^{\text {2}}{\tilde{c}}. \end{aligned}$$

Then, the functions $(a_{ij})$ satisfy (9.5), and the functions $(b_{i})$, *c* satisfy (i) of Theorem 33.

In the next proposition we give sufficient conditions under which the solution is not locally integrable.

### Proposition 35

Let $\Omega \subseteq \mathbb {R}^{d}$ be a bounded open set, $v\in {\mathcal {C}}^{2,*}(\Omega )$ be a map satisfying (6.1). Let $f\in X_{|\cdot |,v,0}^{1}(\Omega )$ be such that (9.2) holds where

(i) $$\forall i,j=1,...,d:\,a_{ij}\in {\mathcal {C}}^{1}(\Omega )\cap L_{v^{-4}}^{\infty }(\Omega )$$ and $$\forall \alpha \in \pi _{1}^{0}:\,\partial ^{\alpha }a_{ij}\in L_{v^{-3},\textrm{loc}}^{\infty }(\Omega )$$
(ii) $$\forall i=1,...,d:\,b_{i}\in {\mathcal {C}}^{1}(\Omega )\cap L_{v^{-3}}^{\infty }(\Omega )$$ and $$\forall \alpha \in \pi _{1}^{0}:\,\partial ^{\alpha }b_{i}\in L_{v^{-2},\textrm{loc}}^{\infty }(\Omega )$$
(iii) $$c\in L_{v^{-2}}^{\infty }(\Omega )$$
(iv) the map *h* is defined by $h(g):=\int _{\Omega }kg\textrm{d}x$, where $k\in L_{v^{-1}}^{2}(\Omega )$, $k\ge 0$ $\mathrm {a.e.}$ in $\Omega$ and $kv^{-2}\not \in L_{\textrm{loc}}^{1}(\Omega )$

Then, $$f\not \in L_{\textrm{loc}}^{1}(\Omega )$$.

### Proof

Use (9.2) with $g=\varphi \chi _{n}v^{-2}\in {\mathcal {C}}_{c}^{2}(\Omega )$ (because supp$(\chi _{n})\subseteq M_{2n}^{c}$) where $\varphi \in {\mathcal {D}}(\Omega )$ and $(\chi _{n})$ is the sequence of ${\mathcal {C}}^{2}(\Omega )$ functions constructed in the proof of Proposition 7 we get

9.12 $$\begin{aligned}  &   \sum _{i,j=1}^{d}\int _{\Omega }a_{ij}D_{v}^{i}f\partial _{j}(\varphi \chi _{n}v^{-2})\,\textrm{d}x+\sum _{i=1}^{d}\int _{\Omega }b_{i}\varphi \chi _{n}v^{-2}D_{v}^{i}f\,\textrm{d}x\nonumber \\  &   \quad +\int _{\Omega }cf\varphi \chi _{n}v^{-2}\,\textrm{d}x=\int _{\Omega }k\varphi \chi _{n}v^{-2}\,\textrm{d}x \end{aligned}$$

Using the weak derivative in the sense of $L_{v,\textrm{loc}}^{1}(\Omega )$ with $a_{ij}v^{-2}\partial _{j}(\varphi \chi _{n}v^{-2})\in {\mathcal {C}}_{c}^{1}(\Omega )$ we get

9.13 $$\begin{aligned} \int _{\Omega }a_{ij}D_{v}^{i}f\partial _{j}(\varphi \chi _{n}v^{-2})\,\textrm{d}x=-\int _{\Omega }f\partial _{i}(a_{ij}\partial _{j}(\varphi \chi _{n}v^{-2}))\,\textrm{d}x \end{aligned}$$

Similarly, using the weak derivative in the sense of $L_{v,\textrm{loc}}^{1}(\Omega )$ with the test function $b_{i}\varphi \chi _{n}v^{-4}\in {\mathcal {C}}_{c}^{1}(\Omega )$ we get

9.14 $$\begin{aligned} \int _{\Omega }b_{i}\varphi \chi _{n}v^{-2}D_{v}^{i}f\,\textrm{d}x=-\int _{\Omega }f\partial _{i}(b_{i}\varphi \chi _{n}v^{-2})\,\textrm{d}x \end{aligned}$$

Using (9.13) and (9.14) in (9.12) we get

9.15 $$\begin{aligned}  &   -\sum _{i,j=1}^{d}\int _{\Omega }f\partial _{i}(a_{ij}\partial _{j}(\varphi \chi _{n}v^{-2}))\,\textrm{d}x-\sum _{i=1}^{d}\int _{\Omega }f\partial _{i}(b_{i}\varphi \chi _{n}v^{-2})\,\textrm{d}x\nonumber \\  &   \quad +\int _{\Omega }cf\varphi \chi _{n}v^{-2}\,\textrm{d}x=\int _{\Omega }k\varphi \chi _{n}v^{-2}\,\textrm{d}x. \end{aligned}$$

A simple calculation gives

9.16 $$\begin{aligned} \partial _{i}(a_{ij}\partial _{j}(\varphi \chi _{n}v^{-2}))= &   \chi _{n}(\partial _{i}a_{ij}\partial _{j}(v^{-2}\varphi )+a_{ij}\partial _{ij}(v^{-2}\varphi ))\nonumber \\  &   +\partial _{j}\chi _{n}(v^{-2}\varphi \partial _{i}a_{ij}+a_{ij}\partial _{i}(v^{-2}\varphi ))+\partial _{i}\chi _{n}(a_{ij}\partial _{j}(v^{-2}\varphi ))\nonumber \\  &   +a_{ij}v^{-2}\varphi \partial _{ij}\chi _{n}\qquad \end{aligned}$$

Developing the expression $\partial _{i}a_{ij}\partial _{j}(v^{-2}\varphi )+a_{ij}\partial _{ij}(v^{-2}\varphi )$ we get

$$\begin{aligned}  &   \partial _{i}a_{ij}(-2(\partial _{j}v)v^{-3}\varphi +v^{-2}\partial _{j}\varphi )\\  &   \qquad +a_{ij}(v^{-2}\partial _{ij}\varphi -2(\partial _{j}v)v^{-3}\partial _{i}\varphi -2(\partial _{i}v)v^{-3}\partial _{j}\varphi \\  &   \qquad -2(\partial _{ij}v)v^{-3}\varphi +6(\partial _{i}v)(\partial _{j}v)v^{-4}\varphi ) \end{aligned}$$

By assumption (i)

9.17 $$\begin{aligned} \exists C>0\,\forall n\in \mathbb {N}:\,|\chi _{n}(\partial _{i}a_{ij}\partial _{j}(v^{-2}\varphi )+a_{ij}\partial _{ij}(v^{-2}\varphi ))|\le C1_{V} \end{aligned}$$

where $1_{V}$ is the characteristic function of the set $V:=\textrm{supp}(\varphi )$. Note now that, for all $\alpha \in \pi _{2}^{0}$ we have $\partial ^{\alpha }\chi _{n}\equiv 0$ on $M_{n}^{c}$ (because by $\chi _{n}\equiv 1$ on $M_{n}^{c}$), and that $|\partial ^{\alpha }\chi _{n}|\le Cn^{|\alpha |}$ on $M_{n}$ for all *n* sufficiently large, where $C>0$ is independent of *n*. This is because $M_{n}\Subset \Omega$ (by assumption (6.1)) (and hence $\partial ^{\alpha }v$ is bounded on $M_{n}$), and because $|\eta _{n}^{(k)}|\le Cn^{|k|}$ where the constant $C>0$ depends on *k* but not on *n*. It follows that

9.18 $$\begin{aligned} \exists C>0\,\exists n_{0}\in \mathbb {N}\,\forall n\ge n_{0}\,\forall \alpha \in \pi _{2}^{0}:\,|v^{|\alpha |}\partial ^{\alpha }\chi _{n}|\le C \end{aligned}$$

since $|v|\le 1/n$ on $M_{n}$. Thus, one can easily prove (as above), using assumption (i) that

9.19 $$\begin{aligned} \left| \partial _{j}\chi _{n}(v^{-2}\varphi \partial _{i}a_{ij}+a_{ij}\partial _{i}(v^{-2}\varphi ))+\partial _{i}\chi _{n}(a_{ij}\partial _{j}(v^{-2}\varphi ))+a_{ij}v^{-2}\varphi \partial _{ij}\chi _{n}\right| \le C1_{V}.\nonumber \\ \end{aligned}$$

for all $n\ge n_{0}$. Assume by contradiction that $f\in L_{\textrm{loc}}^{1}(\Omega )$. Then, by (9.16), (9.17) and (9.19) we have

9.20 $$\begin{aligned} \forall n\ge n_{0}:\,\left| \sum _{i,j=1}^{d}\int _{\Omega }f(\partial _{i}(a_{ij}\partial _{j}(\varphi \chi _{n}v^{-2})))\,\textrm{d}x\right| \le C||f||_{L^{1}(V)}. \end{aligned}$$

By (ii) and (iii), and using similar arguments as above one can prove that

9.21 $$\begin{aligned} \forall n\ge n_{0}:\,\left| \sum _{i=1}^{d}\int _{\Omega }f\partial _{i}(b_{i}\varphi \chi _{n}v^{-2})+fc\varphi \chi _{n}v^{-2}\,\textrm{d}x\right| \le C||f||_{L^{1}(V)} \end{aligned}$$

We deduce from (9.15), (9.20), (9.21) that

$$\begin{aligned} \exists C>0\,\forall n\ge n_{0}:\,\int _{\Omega }k\varphi \chi _{n}v^{-2}\,\textrm{d}x\le C||f||_{L^{1}(V)} \end{aligned}$$

Let now $K\Subset \Omega$ be such that $kv^{-2}\not \in L^{1}(K)$ and choose $\varphi$ so that $\varphi \ge 0$ on $\Omega$ and $\varphi \equiv 1$ on *K*. It follows that $\int _{K}k\chi _{n}v^{-2}\,\textrm{d}x\le C$ for all $n\ge n_{0}$, which leads to a contradiction when choosing *n* sufficiently large since

$$\begin{aligned} \lim _{n\rightarrow \infty }\int _{K}k\chi _{n}v^{-2}\,\textrm{d}x=\int _{K}kv^{-2}\,\textrm{d}x=\infty \end{aligned}$$

by the monotone convergence theorem. $\square$

### Remark 36

If

$$\begin{aligned} \forall i,j=1,...,d:\,a_{ij}={\tilde{a}}_{ij}v^{4},\,b_{i}={\tilde{b}}_{i}v^{3} \end{aligned}$$

where

$$\begin{aligned} \forall i,j=1,...,d:\,{\tilde{a}}_{ij}\in {\mathcal {C}}^{1}(\Omega ),\,{\tilde{b}}_{i}\in {\mathcal {C}}^{1}(\Omega ), \end{aligned}$$

then assumptions (i), (ii) of the latter proposition hold.

### Example 37

For any integer $m\ge 1$, and for any $\beta \in (d/2-2m,d/2)$, the boundary-value problem

$$\begin{aligned} {\left\{ \begin{array}{ll} -\sum _{i=1}^{d}D_{i}(|x|^{8m}D_{i}f)+|x|^{4m}f=|x|^{-\beta +2m} &  \text {in }\{x\in \mathbb {R}^{d}\mid |x|<1\}\\ f=0 &  \text {on }\{x\in \mathbb {R}^{d}\mid |x|=1\} \end{array}\right. } \end{aligned}$$

has a unique non-locally integrable solution $f\in X_{|\cdot |,|x|^{2\,m},0}^{1}(\Omega )$ thanks to Theorem 33 and to Proposition 35.

## Footnotes

1 So that the trace operator on $W^{1,p}(\Omega )$ is well defined.
